# “Old and new” frontiers in embryo viability assessment: current practices and introduction of emerging approaches

**DOI:** 10.1093/biolre/ioag147

**Published:** 2026-07-14

**Authors:** Sareena Rayne Singh, Madeline Abass, Jakub Bartlewski, Joanna Kochan, Maciej Murawski, Jakub Wyroba, Pawel M Bartlewski

**Affiliations:** Department of Biomedical Sciences, Ontario Veterinary College, University of Guelph, 50 Stone Road East, Guelph, Ontario, N1G 2W1, Canada; Department of Biomedical Sciences, Ontario Veterinary College, University of Guelph, 50 Stone Road East, Guelph, Ontario, N1G 2W1, Canada; Department of Clinical Studies, Ontario Veterinary College, University of Guelph, 50 Stone Road East, Guelph, Ontario, N1G 2W1, Canada; Lesser Poland Institute for the Diagnosis and Treatment of Infertility—KrakOvi, Cracow, Poland; Department of Animal Reproduction, Anatomy and Genomics, University of Agriculture in Kraków, Cracow, Poland; Department of Animal Biotechnology, University of Agriculture in Kraków, Cracow, Poland; Lesser Poland Institute for the Diagnosis and Treatment of Infertility—KrakOvi, Cracow, Poland; Fertility Disorders Clinic, Andrzej Frycz Modrzewski Krakow University (UAFM), Kraków, Poland; Department of Biomedical Sciences, Ontario Veterinary College, University of Guelph, 50 Stone Road East, Guelph, Ontario, N1G 2W1, Canada

**Keywords:** Assisted reproductive technologies, embryo viability, preimplantation genetic testing for aneuploidy, algorithmic image analysis, image-based assessment

## Abstract

Reliable assessment of embryo viability remains a critical yet challenging component of assisted reproductive technologies (ART). Traditional methods, including morphological grading and preimplantation genetic testing for aneuploidy (PGT-A), are either subjective, invasive, or resource intensive. Consequently, non-invasive approaches, including analysis of spent culture medium (SCM) and digital image-based analytical methods, emerged as promising alternatives to support clinical decision-making, though their reliability varies. SCM analyses can detect embryonic DNA and metabolites, but their outcomes are constrained by potential contamination with non-embryonic material and inconsistent amplification efficiency. In contrast, image-based assessment combined with machine learning (ML) enables quantitative, repeatable, and transparent evaluation of embryo architecture. Segmental image analysis of grayscale bitmaps allows for extraction of interpretable phototextural metrics, which have been correlated with developmental competence and ploidy status of embryos. Recent applications of ML-assisted techniques demonstrate comparable or superior predictive performance relative to PGT-A and expert embryologists, highlighting their potential as cost-effective and ethically favorable alternatives. Continued refinement of image acquisition minimizing artifacts, optimizing quality and resolution, and focusing on key regions such as the trophectoderm and inner cell mass, may further enhance their diagnostic sensitivity and specificity. Overall, computerized assessment of embryo microphotographs represents a scalable, non-invasive strategy that can complement or even partially replace invasive testing. By leveraging structured, interpretable image data, algorithms such as r-Algo 2.0 (discriminative image-analysis software) exemplify the emerging role of computational tools in advancing diagnostic precision and objectivity in ART.

**Highlights**
 

- Current methods of embryo viability assessment lack clinical reliability
- Non-invasive image analysis supports accurate embryo quality assessment
- Transparent algorithms enhance repeatability of image-analysis techniques
- Image-based embryo assessments may complement or reduce the need for PGT-A
- Imaging refinements can further enhance its diagnostic sensitivity and specificity

## Teaser

Computerized image analysis and machine learning offer a non-invasive, scalable, and reliable alternative to currently used embryo assessment methods, with comparable or improved predictive performance, enhancing objectivity and clinical decision-making in ART.

## Introduction

### Infertility and assisted reproductive technologies in the general population

According to estimates from the World Health Organization, approximately one in six individuals worldwide are affected by infertility [1]. Infertility is defined as the inability to conceive after 12 months of regular, unprotected sexual intercourses; for women aged 35 years or older, this period is reduced to 6 months due to the natural decline in fertility with age. To combat infertility and improve reproductive outcomes, both the availability and efficacy of assisted reproductive technologies (ART), such as in vitro fertilization (IVF) and intracytoplasmic sperm injection (ICSI), must continue to advance. However, recent reports from the Society for Assisted Reproductive Technology (SART) indicate that ART success rates remain modest and decrease with age, with live birthrates, for new patients whom started their ART procedure(s) within the reporting year, averaging 50% for women under the age of 35, ~33% for women aged 35–40, and 14% for women aged 41-42, and 4% for women over the age of 42 [2]. Consequently, infertility and the pursuit of ART can impose substantial financial, psychological, and emotional burdens on affected individuals and couples [3, 4]. Identifying and implementing methods that improve ART is imperative not only for increasing the success of childbearing (clinical perspective), but also for reducing the hardships associated with ART (patients’ perspective).

To improve ART success rates by increasing the likelihood of implantation, clinical embryologists may choose to transfer more than one embryo. Although this can increase live birth rates (LBRs), it also elevates the risk of impending health complications. Therefore, it is generally recommended that clinics transfer a single embryo per IVF cycle, depending on the patient’s age and underlying cause of infertility [5, 6]. To enhance the success of IVF with single embryo transfer, fertility clinics seek to identify and select a transferrable embryo with the highest developmental potential. The development of accurate and reliable techniques for evaluating embryo viability has become essential for improving the overall efficacy of contemporary ART.

Current approaches to determining embryo viability mainly include morphological assessment and preimplantation genetic testing for aneuploidy (PGT-A; [8]). Standard non-invasive approaches, such as morphological assessment, are relatively simple and accessible, but inherently subjective and prone to variability [8]. In contrast, PGT-A provides an objective evaluation of an embryo’s chromosomal status; however, it is costly and can be damaging to the embryo [9, 10]. Paulson [11] reported that PGT-A may reduce pregnancy rates due to its potential adverse effects. As a result, PGT-A is only recommended for individuals with a history of failed embryo transfers/recurrent miscarriages [7, 11]. Evidently, non-invasive methods for assessing embryo viability eliminate many of the risks associated with PGT-A. Consequently, there is growing interest in developing more affordable and reliable non-invasive methods of evaluating the developmental potential of in vitro–derived embryos, and such strategies are currently the focus of increasingly intensive research (Figure 1). The “omics” techniques, including transcriptomics, proteomics, and metabolomics, are being extensively studied to identify measurable biomarkers in the embryos’ spent culture media (SCM) [8]. Time-lapse imaging (TLI) enables continuous visual assessment of an embryo’s morphological changes, providing important insights into potential morphokinetic differences between embryos varying in their developmental potential [8]. Furthermore, artificial intelligence (AI), particularly machine learning (ML) algorithms, represents one of the most promising approaches for improving ART by predicting embryo viability based on multiple quantifiable parameters [12]. In addition, emerging non-invasive approaches, such as ultra-weak photon emission (UPE), which measures natural luminosity produced from metabolic and physiological processes, and algorithmic phototextural image analysis focusing on previously unexplored intrinsic attributes of developing embryos, are currently under active investigation and are rapidly progressing from the proof-of-concept to validation stages.

**Figure 1:**
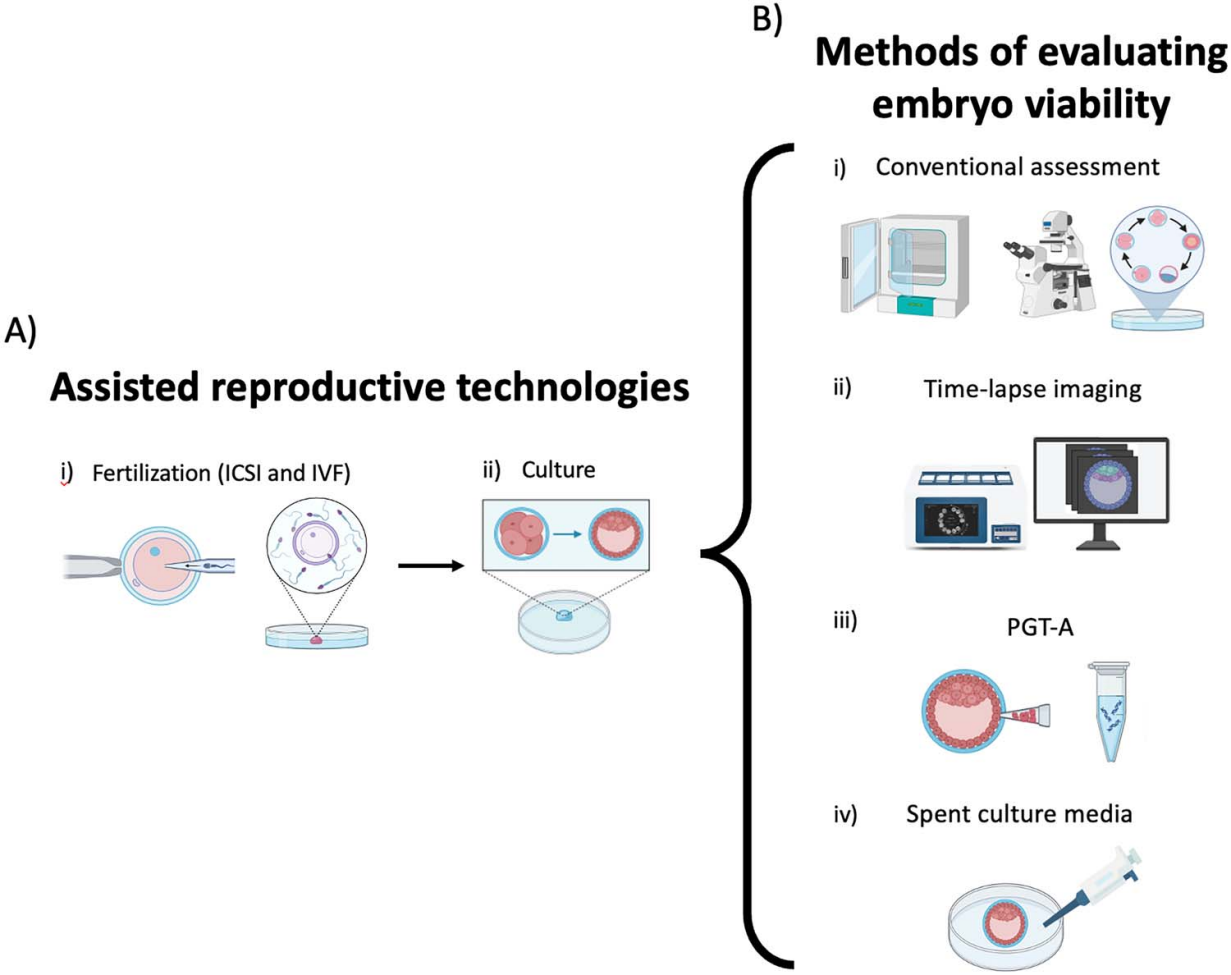
A schematic of different methods of embryo viability assessment. (A) Fertilization is achieved through assisted reproductive technologies (ART)-in vitro fertilization (IVF) or intracytoplasmic sperm injection (ICSI). (B) A schematic of the different embryo grading assessment methodologies including (i) conventional assessment where an embryo is removed from an incubator and examined under a microscope at different stages of its development; (ii) PGT-A with laser excision of 5–10 cells from the trophoblast of an embryo at day 5–6 post-fertilization; (iii) time-lapse imaging (TLI) assessment of an embryo as it develops in a closed incubator via taking images every 5–15 min, to collect morphometric and developmental data; (iv) spent culture media (SCM) assessment through using media collected either after embryo transfer (one-step medium) or at different developmental stages (multi-step media).

This review summarizes current clinical methods of embryo viability assessment and introduces, in more depth, a novel non-invasive technique utilizing the algorithm-based quantitative image analysis of embryo microphotographs, which may offer a means of inferring embryo ploidy status. A comparative evaluation of the advantages and limitations of both the established techniques and new methodologies is also presented.

## Current clinical methods

### Morphological assessments

The primary method for assessing embryo viability involves removing embryos from an incubator to examine their morphological features under a microscope. The morphology and structural appearance of the embryo provide important indicators of its health status. A fertilized oocyte, or presumptive zygote, is classified as being in the pronuclear (PN) stage when it remains a single cell. During this stage, the nuclei of the sperm and oocyte fuse to form a single nucleus. The zygote then begins to divide, marking the onset of the cleavage stage. By days 5–6 post-insemination, the human embryo develops into a blastocyst consisting of approximately 50–200 cells.

#### Nuclear stage morphological assessment

The most assessed characteristics at the PN stage of embryonic development include the size of the nucleoli, distribution of nucleolar precursor bodies (NPBs) within the pronuclei, and their overall symmetry. NPBs are distinct, electron-dense, spherical nuclear structures present in the oocytes and early embryos of mammalian species. They represent immature forms of the nucleolus and play a significant role in the re-establishment of functional nucleoli after fertilization. There are several methods to distinguish between normal and abnormal presumptive zygotes. One widely used PN grading scheme, developed by Tesarik and Greco [13], evaluates the number, relative size, and location of NPBs within the male and female pronuclei. This scheme comprises six patterns (0–5) that represent progressive stages of PN development and symmetry (Supplementary Figure 1). In general, the transition from pattern 0 (early or unorganized stage) to pattern 1 (symmetrical alignment) reflects normal pronuclear maturation, whereas increasing irregularity from patterns 2 through 5 (slight, moderate, and marked asymmetry, potentially culminating in complete disorganization) indicates declining zygote quality. Scott [14] proposed a PN grading system that assigns a Z-score to embryos based on the halo, alignment, and number of pronuclei (Supplementary Figure 2). Due to the nature of these scoring methods being highly thorough and therefore time-consuming, they are impractical for routine use in a laboratory setting. A retrospective study of >3000 zygotes by Stigliani et al. [15] demonstrated that applying a simplified PN grading scheme developed by the European Society of Human Reproduction and Embryology (ESHRE; [16]) could also improve the accuracy of embryo viability assessment; the ESHRE system classifies NPBs into three categories or scores: (1) symmetrical, (2) non-symmetrical, and (3) abnormal (Supplementary Figure 3). When scores 2 and 3 were combined for analysis, they found the significantly different 15% implantation rate for score 1 embryos and 9% implantation rate for PN score 2–3 embryos. Importantly, consistent with earlier studies [17, 18], this study did not confirm the predictive value of optimal PN morphology for post-implantation development or long-term pregnancy outcomes. Lastly, the time window for PN assessment is very narrow (approximately 17 ± 1 h post-ICSI), after which the pronuclei are no longer discernible, further limiting its practicality [16].

#### Cleavage-stage morphological assessment

Human embryos reach the cleavage stage approximately 2 days after insemination and are assessed on days 2–3. Day 3 of development is singularly important because this is when embryonic genome activation takes place and many embryos arrest at this stage (https://obgynkey.com/chapter-8-timing-of-embryo-culture/; last accessed March 2026). The main cleavage-associated morphological features evaluated include cell number, symmetry, and the degree of fragmentation [19]. There are different methods that have been developed to assess cleavage-stage embryo morphology [19–21]. In practice, however, embryo viability at the cleavage stage is often evaluated using customized or clinic-specific systems. For instance, one Canadian clinic classifies cleavage-stage embryos on a scale of A–C, with grade A embryos having ≤10% fragmentation (https://www.onefertilitykitchenerwaterloo.com/ivf-embryo-grading-guide/; last accessed: March 2026); whereas another clinic will classify cleavage-stage embryos on a scale of 1–4, based on cell size and fragmentation, with grade 1 characterized by evenly sized cells and lower fragmentation being of higher quality (https://www.arcfertility.com/understanding-embryo-grading/; last accessed: March 2026). Most of the clinics, however, use a set of criteria similar to the grading scale developed by Stensen et al. [21] (Figure 2).

**Figure 2:**
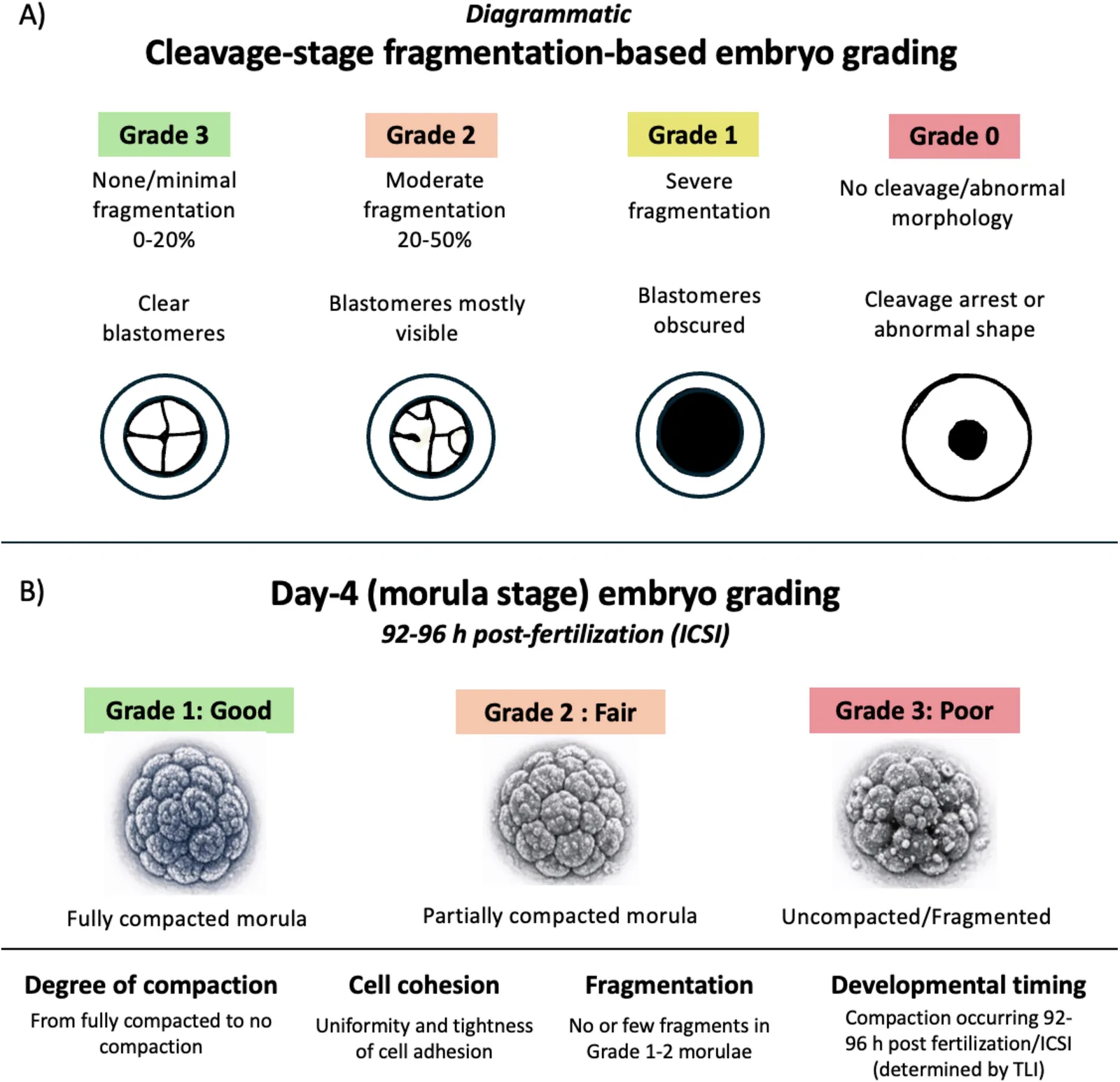
A schematic of embryo grading at the cleavage and morula stage of embryonic development. (A) Cleavage-stage embryo grading system for estimating embryo viability and developmental potential developed by Stensen et al. [21]. (B) Visual principles of embryo grading at the morula stage [16].

The morphological grading at the cleavage stage has been shown as a good predictor of embryo development to the blastocyst stage, but not of blastocyst transfer outcome or fate [22, 23]. A retrospective study by Zilberberg et al. [24] showed that if when evaluating the developmental progression of an embryo that turned into an excellent quality blastocyst, its appearance at the cleavage stage did not affect ongoing pregnancy rates (typically defined as pregnancy beyond the first trimester). More precisely, comparing cleavage-stage morphology between good-quality blastocysts is not advantageous for predicting transfer outcome.

Interestingly, there is evidence that the morphological appearance of a cleavage-stage embryo in conjunction with cleavage timing may be more accurate in determining embryo quality [25, 26]. Studies have shown that embryos that cleave early have a higher pregnancy rate than late-cleavage embryos [27–29]. However, this assessment can only be completed with the use of TLI systems that monitor and determine the “tempo” at which individual embryos cleave. Overall, embryo development at the cleavage stage is a marker of subsequent blastocyst development and quality but remains a variable predictor of pregnancy following the transfer of pre-selected blastocysts. Thus, cleavage-stage assessment alone is not a sufficient measure of embryo viability.

#### Day-4 human embryo assessment

After cleavage, day 4 of human embryo culture marks the attainment of the morula stage. Most normally developing embryos contain ~12–16 cells by day 4 post-insemination/ICSI. At this point, embryos are assessed visually to estimate their developmental progress on route to the blastocyst, including cell number, compaction, overall symmetry, and the degree of fragmentation (Figure 2). The Istanbul Consensus (Alpha/ESHRE; [16, 30]) explicitly describes day-4 embryo evaluation as descriptive, not numeric ranking. The relative predictive value of day-4 embryo assessment in terms of ensuing implantation and pregnancy rates remains low to moderate. Although a retrospective analysis of graded day-4 embryos found significant correlations with pregnancy rates [31], most studies indicate that grading at day 4 is better *as a screening tool* for culture continuation rather than for direct prediction of pregnancy outcome [31, 32].

#### Blastocyst-stage morphological assessment

Most clinics transfer embryos between days 5 and 6 post-fertilization. The practical and most widely used grading system for assessing embryos just prior to transfer, developed by Gardner and Schoolcraft [33], evaluates three distinctive morphological components of the blastocyst: the trophectoderm (TE), the blastocoel (fluid-filled cavity), and the inner cell mass (ICM; Supplementary Figure 4). Because most clinics employ conventional assessment methods (i.e. removing embryos from incubators for morphological evaluation), the Gardner and Schoolcraft [33] system remains the standard approach for blastocyst assessment. Using this grading scheme, Gardner et al. [34] reported that the implantation rate (defined by the presence of a fetal heartbeat) of individuals who underwent the transfer of a single high-scoring blastocyst resulted in a 50% implantation rate. Furthermore, Alfarawati et al. [35] demonstrated that aneuploid embryos received lower grades than euploid embryos, particularly in the ICM and TE regions. These findings have contributed to improved success rates of single embryo transfer (SET) procedures [36–39].

Although higher blastocyst scores are associated with improved implantation and pregnancy outcomes, this assessment has notable limitations. Most importantly, it is based on subjective visual evaluations by embryologists, which can vary depending on individual experience. Storr et al. [40] compared the grading and selection of day-5 blastocysts among 10 embryologists performing SET and found that, among 100 cases, the 10 embryologists agreed on the same (top) embryo for transfer in only 50% of cases. Inter-observer variability increases even further when embryos are evaluated at multiple discrete time points rather than just one [40, 41]. In addition, the association between good-quality morphology and euploidy declines with advancing maternal age, which could increase inter-observer agreement [42, 43].

Collectively, despite extensive research showing how morphological grading of blastocysts has significantly improved clinical pregnancy rate (CPR) and live birth rates (LBRs), compared to that of PN and cleavage-stage assessment, blastocyst grading is a subjective and inconsistent method for assessing embryo viability. However, blastocyst scoring currently remains the single most practical and successful method at selecting the best embryo for transfer.

### Preimplantation genetic testing

Since morphological grading criteria do not fully account for the genetic composition of an embryo, frequently resulting in implantation failure, miscarriage, or genetic abnormalities in offspring [44, 45], Handyside et al. [46] introduced preimplantation genetic testing (PGT). Initial applications of PGT were geared toward preventing X-linked diseases in affected families [44, 45]. This was followed by work at the Jones Institute in the United States, which in 1995 performed PGT to detect Tay–Sachs disease, a fatal genetic neurodegenerative disease, leading to the birth of the first healthy baby following PGT [47]. Currently available PGT diagnostics include testing for structural rearrangements (PGT-SR), monogenic diseases (PGT-M), and aneuploidy (PGT-A) [44]. For the purposes of this review, only PGT-A is discussed.

Aneuploidy typically originates from errors during meiotic division such as non-disjunction (chromosomal mis-segregation; [48, 49]). Meiotic errors largely produce whole-embryo aneuploidy, in which every cell is chromosomally abnormal. However, aneuploidy can also arise from mitotic errors, which occur after fertilization and produce predominantly mosaic embryos, in which the cumulative cell population has differing cell lines, including both aneuploid and euploid cells that may be clustered or dispersed [50–52]. A typical TE biopsy sample collects only 5–10 cells from the blastocyst which is analyzed using next-generation sequencing (NGS) [53]. However, the probability those cells failing to represent the chromosomal status of the entire embryo can be substantial. This can be mathematically visualized with the binomial sampling equation: ${\left(1-p\right)}^n$ where *p* is the percentage of cells that are aneuploid and *n* is the number of cells collected through biopsy. For example, if aneuploid cells constitute 20% of the embryo, the probability that PGT-A sampling detects none is approximately 33%. When the aneuploid proportion is 10%, the probability of missing aneuploid cells increases to about 60%. If the aneuploid cell content is very low (<5%), the probability of non-detection can reach 80–90%.

The main challenge with embryonic mosaicism lies in its unspecified biological and clinical interpretation. Mosaicism detected by PGT-A may not accurately represent the chromosomal status of the entire embryo [53, 54]. As a result, the true level of mosaicism is often unknown, leading many clinics to adopt a simplified binary classification (euploid vs. aneuploid) system despite its limitations [54]. Clinically, there is ongoing debate about whether mosaic embryos, particularly those with low-level mosaicism, should be reported or transferred. While professional guidelines (e.g. ESHRE) and previous studies indicate that the transfer of low- to medium-level mosaic embryos is associated with implantation rates and neonatal outcomes comparable to those for euploid embryos [52, 55, 56], other recent evidence suggests a modest but significant reduction in implantation rates [57, 58]. These conflicting findings highlight the need for standardized reporting criteria and transfer guidelines of mosaic embryos, as well as cautious clinical decision-making.

PGT-A is an invasive procedure that requires substantial technical skill, dexterity, and experience. The current methodology involves an embryologist performing a TE biopsy on day-5–6 blastocysts using a laser to excise TE cells. The isolated cells undergo whole-genome amplification (WGA) followed by NGS to determine the embryo’s ploidy status. The entire workflow, from TE biopsy to ploidy reporting, typically spans 12–48 h, depending on the WGA and NGS platforms used. Therefore, this process necessitates vitrification, warming, and subsequent transfer of assessed blastocysts. Bhatt et al. [59] looked at the effect of PGT-A on pregnancy outcomes in patients with recurrent pregnancy loss (RPL), which they defined as three or more pregnancy losses in autologous frozen embryo transfer cycles. Among embryos subjected to PGT-A, there was a significant improvement in LBR (47.7%) and a reduction in adverse outcomes, including ectopic pregnancy (19.0%) and spontaneous abortion (10.8%). The most pronounced benefit was observed in patients aged >40 years, a group with a higher incidence of aneuploidy and RPL. These findings align with more recent data from Harris et al. [60] who reported that individuals aged ≥35 years who underwent PGT-A had a higher CPR (54.3–64.8%), defined by the presence of a positive pregnancy past the first trimester, compared with those who did not (33.7–54.5%). Overall, across all age groups, patients who utilized PGT-A were approximately 3% more likely to achieve a live birth [60].

Despite its benefits, PGT-A remains an invasive procedure, as it involves removing TE cells during ongoing embryonic development, which can significantly alter the embryo’s architecture. The removal of TE cells during PGT-A may be associated with adverse outcomes, including implantation failure [7]. A recent retrospective study found that technical biopsy errors, amplification failures, and diagnostic inaccuracies may result in only 24.4% of biopsied embryos being eligible for transfer [61]. In addition to its biological risks, PGT-A is costly and hence, often inaccessible to many patients. Davis et al. [7] conducted a cost–benefit analysis in a Canadian fertility clinic across different maternal age groups. With the procedural costs approximating CAD $2500 per embryo, their findings indicated that PGT-A provided a clear benefit only for patients over the age of 38, due to a higher prevalence of meiotic errors and a lower number of fertilized oocytes in this age group [7]. While PGT-A is imperative for patients of advanced maternal age, it is still recommended more broadly to increase pregnancy success rates [7]. However, although PGT-A may improve the likelihood of transferring a viable embryo, it does not guarantee a positive outcome, which can sometimes be misconstrued in patient counseling.

Another limitation of PGT-A is that it is typically not performed on poor-quality blastocysts. Good morphological quality does not guarantee chromosomal normality, meaning that morphologically high-quality embryos may still be aneuploid and vice versa, while some lower-quality embryos may be euploid and capable of resulting in healthy pregnancies. Therefore, excluding embryos from PGT-A testing or transfer based solely on morphology may lead to the discarding of potentially viable embryos.

PGT-A is not authorized or uniformly regulated in all countries, largely due to ethical, legal, and societal concerns, particularly around embryo selection and concerns about eugenics [62]. As a result, access to PGT-A varies considerably across jurisdictions [54]. For example, in Germany PGT-A is highly restricted under the Embryo Protection Act and is generally permitted only in exceptional cases (e.g. high risk of severe genetic disease) [62]. In several European states, PGT-A is allowed but strictly regulated (e.g. in Austria, Switzerland, Italy, and France) [62]. Accordingly, scientists provide guidance to embryologists on selecting non-PGT-A embryos for transfer to improve the procedure’s success rates (e.g. prioritizing a small hatching blastocyst over a larger, non-hatching one) [63]. In addition, these regulatory disparities have contributed significantly to fertility tourism [62, 64]. While this expands patient choice, it also raises concerns about inequitable access, uniformity of clinical standards, legal uncertainty regarding embryo ownership, and continuity of medical care and overall cost [62, 64].

Although PGT-A provides an objective estimate of embryo viability, it is not the most optimal method for clinical assessment. Due to the methodological, ethical, and financial challenges associated with PGT-A, considerable attention has shifted toward the development of non-invasive assessment approaches that are more secure, cost-effective, and sufficiently accurate for evaluating embryo viability.

## Non-invasive methods

The advent of TLI technology has enabled continuous, minimally disruptive monitoring of embryos across multiple developmental stages [41, 65, 66]. Application of TLI has revealed the temporal differences that may help identify embryos with greater developmental potential in vitro [66]. Another non-invasive approach involves analyzing the SCM to identify measurable biomarkers of embryo viability. Secretome profiling, the assessment of molecules released by the embryo into the culture medium, integrates several “omics” methodologies, including genomic (nuclear and mitochondrial DNA), metabolomic (small molecules such as sugars and amino acids), and transcriptomic (microRNA) analyses. More recently, AI has emerged as a complementary tool that integrates visual, morphokinetic, and clinical data to predict the most viable embryo for SET. Together, these strategies represent the current landscape of non-invasive assessments, with the potential to improve accuracy while reducing procedural risks of presumptive zygote and embryo grading.

### Spent culture media analysis

Spent medium contains various products secreted by the embryo, including proteins, energy metabolites, and cell-free DNA (cfDNA; [67, 68]). These molecules are aspirated from the culture medium and analyzed using a range of biochemical amplification techniques to characterize the embryo’s secretome profile [69, 70]. The medium can be aspirated at different time points from either single-step (continuous) embryo culture systems, in which the medium is not changed during the entire in vitro embryo culture (IVC) period, or sequential multi-step culture systems, where the medium is changed at different stages of development. Several analytical approaches have been developed to study the secretome, including metabolomics, genomics, and transcriptomics.

#### Metabolomics

Vital developmental events during embryogenesis lead to changes in both the utilization of metabolic products and their release into the culture medium [68]. For instance, as the embryo progresses to the cleavage stage, pyruvate and glutamine serve as the primary energy substrates; however, at the blastocyst stage, glucose becomes the predominant energy source [71–73]. Metabolomics is an “omics” approach that measures metabolites, defined as small molecules (typically <1500 Da and often <1000 Da; [74]). These include various amino acids (e.g. glycine, alanine), organic acids (e.g. lactate, citrate), sugars and sugar alcohols (e.g. glucose, sorbitol), lipids and fatty acids (e.g. phospholipids, triglycerides), nucleotides and nucleosides (e.g. ATP, adenosine), and vitamins or cofactors (e.g. NADH, coenzyme A). Renard et al. [75] were the first to study the association between embryo viability and metabolite profiles in bovine in vitro produced systems, demonstrating that blastocysts with higher glucose uptake exhibited improved developmental and greater implantation success. Similar trends have been observed in human studies, where increased glucose uptake correlated with improved embryo transfer outcomes [76, 77].

Differences in protein turnover between embryos of differing ploidy status have been reported, likely due to the effects of ploidy on mitochondrial activity [78]. A study by Miao et al. [79] found that aneuploid embryos, including those of poor morphological quality, had higher glutamine consumption rates than euploid embryos. Similarly, a study by Sánchez-Ribas et al. [80] used nuclear magnetic resonance (NMR) spectroscopy to compare the metabolic signature between aneuploid and euploid embryos. Their analysis showed a decrease in the level of isoleucine in trisomy 21 embryos (pure and partial trisomy 21) compared to monosomy 21 and normal embryos, suggesting that differences in the metabolic profiles of embryos can distinguish chromosomal abnormalities. Metabolic profiles can also be influenced by embryo sex, as research has shown female embryos to exhibit higher rates of glucose consumption than male embryos [76].

However, conflicting findings in the analysis of embryonic metabolic profiles limit the clinical applicability and widespread use of SCM analysis in ART. For instance, some studies have shown that the SCM profile of good-quality day-3 embryos showed decreased glutamine consumption compared with that of poor-quality embryos [79, 81]. Conversely, another study has reported that glutamine depletion in the SCM of day-3 embryos is associated with successful implantation [82]. These discrepancies may be attributed to differences in the analytical techniques used to assess metabolites, such as liquid chromatography and NMR spectroscopy. Nevertheless, methods such as mass spectrometry is costly and remains difficult to implement in routine clinical practice [83]. Therefore, the identification of reliable metabolic markers of embryo viability requires further investigation before becoming a reliable biomarker of embryo viability. Future research should aim to standardize measurement methods to achieve consistent associations between the embryonic secretome and implantation outcomes and to decrease their cost.

#### Genomics

Measuring embryonic cfDNA accumulating in SCM offers another non-invasive method of embryo assessment [84]. The mechanisms by which embryonic DNA is released into the culture medium remain unclear. Earlier studies have demonstrated the presence of cfDNA in human blastocoel fluid (BF; [85, 86]), suggesting that BF could serve as a less invasive source for PGT-A than trophoblastic cells [85–87]. These findings have also led researchers to propose that BF-derived cfDNA may enter the culture medium via extracellular vesicles [67, 88, 89]. Alternatively, as the embryo develops, apoptosis of cells within the ICM and TE may contribute to cfDNA accumulation in the medium [67, 90, 91]. Stigliani et al. [84] were the first to detect embryonic cfDNA of both nuclear and mitochondrial origin in SCM. While the importance of proper nuclear genome organization and expression in embryonic development is well established, the role of the mitochondrial genome has recently gained increasing attention [92]. This is largely due to studies showing that embryos of poor morphological quality contain higher amounts of cfDNA, both mitochondrial and nuclear, than embryos with superior morphology [93].

##### Nuclear genome

Assessment of the nuclear genome in SCM represents a critical non-invasive method for determining embryo ploidy status, sex, and the presence of X-linked aberrations [94]. Early investigations confirmed the presence of nuclear DNA in embryo-derived SCM; however, they did not account for DNA present in the SCM of non-embryonic origin [84, 93]. Hammond et al. [95] demonstrated that both SCM and non-spent (fresh) media contained measurable levels of mitochondrial (mtDNA) and nuclear DNA, confirming the presence of non-embryonic DNA in culture systems.

Contamination in the SCM can arise maternally from residual cumulus or granulosa cells, paternally from residual sperm cells from traditional in vitro fertilization (IVF) in the medium, externally from additives such as human serum albumin, and a non-sterile laboratory environment [85, 96]. This is problematic for researchers to evaluate the relationship between cfDNA and clinical outcomes. Another critical limitation of genomic SCM analysis is DNA amplification failure. A study by Hammond et al. [95] measuring the concentration of DNA in SCM reported that SCM from blastocyst-stage embryos contained approximately 4 copies of genomic DNA and 600 copies of mtDNA, with the low nuclear DNA copy number frequently leading to amplification failure.

Several studies have evaluated the concordance between cfDNA in SCM and traditional TE biopsy [88, 91, 97, 98]. Reported concordance rates were moderate and variable, reflecting both biological and technical challenges of both diagnostic methods. While some studies demonstrated reasonable agreement [91, 98], especially for detecting whole-chromosome aneuploidies [97], others reported lower concordance [88, 90]. Differences likely arise from DNA origin heterogeneity (embryonic vs. apoptotic cells), DNA degradation level, and contamination, indicating that SCM results should currently be interpreted with caution and are not yet considered equivalent to TE biopsy [96].

##### Mitochondrial DNA

Mitochondria play a crucial role in embryonic development by producing ATP, regulating calcium signaling, and generating reactive oxygen species, which mediate key cellular processes such as proliferation, differentiation, and apoptosis [99, 100]. Mitochondrial DNA (mtDNA), approximately 16.6 kbp in length, encodes 13 peptides essential for oxidative phosphorylation [99, 100].

During oogenesis, mtDNA replicates extensively, and by metaphase II, oocytes typically contain at least 10^5^ copies of mtDNA [101, 102]. However, mtDNA replication ceases following fertilization and remains halted throughout early preimplantation development. Consequently, fixed mtDNA copy numbers are distributed among dividing blastomeres, and the zygote depends on the maternal mitochondrial genome for energy production until the attainment of the blastocyst stage [100]. Since mtDNA is maternally inherited, its copy number, which is the sum of mitochondrial genomes per cell, can be associated with the reproductive age of the patient and embryo viability [101]. Several studies have reported associations between mtDNA copy number with fertilization potential and embryo viability [102]. Diez-Juan et al. [103] analyzed mtDNA in blastomeres from day 3 embryos and found that euploid embryos of lower quality and lower implantation potential had higher mtDNA content. These findings support the “quiet embryo” hypothesis, which posits that mitochondrial activation and replication increase in response to metabolic stress, reflecting the higher energy demands of less viable embryos [103].

With advancements in sensitive and precise amplification techniques, it has become possible to reliably detect mtDNA in SCM [92, 93, 104, 105]. A study by Stigliani et al. [93] observed that high mtDNA content in the SCM of day-3 embryos was correlated with improved blastocyst quality and increased implantation potential. In contrast, Abdellatif et al. [92] reported that mtDNA content in day-3 SCM was not statistically different between embryos that failed and achieved implantation, highlighting that mtDNA content alone is not a reliable predictor of implantation potential. In addition, several studies have shown that there is no statistically significant relationship between mtDNA content in SCM and blastocyst morphological grading [68, 92, 104]. These conflicting results may be attributed to several factors. For example, Zhang et al. [105] highlighted how previous research using the D-loop region of mtDNA to quantify its copy number is not reproducible as the D-loop region is more susceptible to variation from environmental stressors than other target regions, therefore increasing the potential for differing results. In addition, Abdellatif et al. [92] and Kobayashi et al. [68] indicated that limited sample sizes may reduce the statistical significance of observed trends between SCM mtDNA levels and clinical outcomes. Furthermore, the mechanism of how cell-free mtDNA is secreted into the SCM limits the ability of researchers to associate cfDNA with clinical outcomes. Evidence of embryos having the ability to self-correct and expel abnormal cellular debris weakens the argument that mtDNA content is associated with negative clinical outcomes [106]. There undoubtedly needs to be further investigation into whether mtDNA levels in SCM can serve as a reliable biomarker of embryo viability.

#### MicroRNAs

MicroRNAs (miRNAs) are evolutionarily conserved, small non-coding RNA molecules, approximately 22 nucleotides in length, localized within the cytoplasm [107]. They play crucial roles in regulating gene expression through the repression or degradation of target mRNA sequences. miRNAs released by embryos into SCM have been proposed as non-invasive biomarkers of embryonic gene expression and viability [108]. Abu-Halima et al. [109] reported that elevated levels of miRNA-19b-3p were associated with enhanced early cell proliferation, correlating positively with higher pregnancy rates. Similarly, Wang et al. [110] identified six miRNAs (miR-199a-3p, miR-199b-3p, miR-379-5p, miR-199a-5p, miR-432-5p, and hsa-miR-483-5p) within SCM of day-4–5 blastocysts that were indicative of positive pregnancy outcomes. Additionally, Capalbo et al. [111] demonstrated that increased concentrations of miRNA-20a and miRNA-30c were present in the SCM of euploid embryos. Recent studies analyzing miRNA expression in TE biopsy samples confirmed that SCM from aneuploid embryos contained significantly lower levels of miRNA-30c compared with euploid embryos [112, 113].

Among the currently identified miRNA biomarkers, miRNA-191*,* part of the 191/425 miRNA cluster located on human chromosome 3*,* appears to be one of the most promising indicators of embryo viability. Functionally, miRNA-191 is involved in regulating cell differentiation and fate during early embryogenesis [114]. Several studies have reported significant correlations between SCM miRNA-191 levels, embryo ploidy status, and implantation potential [109, 113–116]. However, Rosenbluth et al. [114] observed that miRNA-191 was expressed at higher levels in SCM of aneuploid embryos and those that failed to implant, whereas Sánchez-Ribas et al. [117] found that miRNA-191-5p was more abundant in the SCM of euploid embryos; this transcript has also been detected at higher levels in embryos that successfully implanted. The inconsistencies among these studies may be attributed to certain methodological differences or the source of culture media. Sánchez-Ribas et al. [117] showcased the presence of non-embryonic miRNA transcripts in fresh culture media, highlighting the potential influence of extraneous contaminants on SCM-based miRNA profiling [115, 118].

### The “omics” approaches in review

To reiterate, the apparent advantages of SCM-based testing include its lower cost relative to traditional biopsy, technical simplicity, non-invasiveness, and applicability to embryos of lower morphological quality, which are typically excluded from TE biopsy. However, there are numerous methodological challenges that limit the clinical applicability of “omics”-based approaches in ART. The limitations of SCM-based testing include the strict requirement for individual embryo culture, its applicability only following ICSI (as SCM obtained after conventional IVF is susceptible to sperm DNA contamination), and the need for extremely thorough oocyte denudation, since even a single residual cumulus (granulosa) cell may result in maternal DNA contamination [96, 97]. In addition, SCM-based testing is associated with higher rates of amplification failure, leading to an increased risk of false-positive and false-negative results compared with TE biopsy [97].

Furthermore, the lack of methodological standardization across studies compromises the reproducibility and validity of SCM-based findings. For instance, the volume of SCM aspirated for miRNA analysis varies substantially between studies. Hammond et al. [95] collected 20 μL, Rosenbluth et al. [114] used 6 μL, and Sánchez-Ribas et al. [117] analyzed 50 μL of sample. Such discrepancies can significantly affect analytical sensitivity by differentially diluting measured metabolites, cfDNA, and miRNAs, leading to misinterpretation or conflicting results. Additionally, sampling at different time points can also alter cfDNA, miRNA, and metabolite concentrations. Galluzzi et al. [119] reported significant differences in nuclear DNA content from SCM between day-3 and day-5–6 embryos. Other confounding factors including media replacement protocols, such as sequential or continuous media, influence the quantity and composition of the embryo secretome, further increasing variability among studies [120].

Commercial manufacturers often withhold full disclosure of media-specific ingredients including protein supplements [120]. In a comprehensive analysis of more than 80 commercial media, Zagers et al. [120] found that no two medium formulations were identical in either composition or ingredient concentrations, including key nutrients such as glucose, proteins, and inorganic ions. Mantikou et al. [121] demonstrated that variations in the source and composition of commercial culture media can directly influence embryo viability. They reported that embryos cultured in Cook Medical “Sydney IVF” medium exhibited lower viability than those cultured in Irvine Scientific “P1” or Vitrolife “G3” media. The industry-wide variability in embryo culture media, coupled with the lack of methodological uniformity and potential for contamination, significantly compromises the reliability of SCM-based “omics” approaches. At present, SCM analysis cannot be regarded as a reliable non-invasive method for assessing embryo viability.

### The advancements of assessing embryo morphology at multiple time points via TLI

Single time-point assessments are inadequate for evaluating embryonic development because embryonic structure and health status can change substantially over short time intervals [41, 65]. The use of TLI technology helps mitigate this limitation by capturing successive images that allow continuous evaluation of embryonic development [122]. A TLI system integrates an incubator with a microscope connected to an external computer, capturing images every 5–15 min to produce a time-lapse sequence of in vitro development [123]. Compared to traditional incubators, TLI incubators minimize the time embryos spend outside controlled culture conditions, thereby reducing exposure to fluctuations in pH, temperature, and gas composition that could negatively affect development [123–125].

The recent introduction of TLI incubators into fertility clinics has enabled embryologists to gather extensive morphokinetic and developmental data, significantly improving the ability to assess embryo viability [26, 124, 126–128]. With the advancement of TLI technology, Dal Canto et al. [129] showed that cleavage-stage embryos that divided more rapidly from the 2–8-cell stage metamorphosed into high-quality blastocysts with improved implantation rates. Previous studies looking at using TLI to predict CPR, which is typically defined by the presence of a positive pregnancy beyond first trimester, in comparison to conventional grading reported that TLI produced a higher CPR [128, 130].

However, evidence showcasing the clinical benefits of TLI compared with conventional morphological assessment is conflicting. For example, Armstrong et al. [125] reported that although TLI reduces the time embryos spend outside the incubator, there is no compelling evidence that it improves embryo viability or clinical outcomes. Similarly, a randomized controlled study by Meng et al. [131] compared embryos cultured from day 2 to day 5 in TLI and conventional incubators, and found no statistically significant differences in embryonic development, implantation rate, or CPR. Another prospective, randomized control trial conducted by Kieslinger et al. [132] compared pregnancy rates of embryos cultured and morphologically assessed in conventional interrupted (control) and uninterrupted TLI incubators. Embryos in the TLI incubator were assessed using two approaches: (1) early embryo viability assessment (TLE) or (2) time-lapse routine morphological grading (TLR). The embryos in the TLE group were assessed using an early embryo viability assessment algorithm in combination with routine uninterrupted morphological grading; the TLR embryos were assessed using only routine uninterrupted morphological grading and the control group was assessed using routine interrupted morphological grading. In all groups, embryos were evaluated using the same criteria (i.e. blastomere number, symmetry, and degree of fragmentation) between days 1 and 3 post-fertilization. The study found no significant differences in CPR or LBR among the TLE, TLR, and control groups [132]. These findings are consistent with other studies that showed no significant advantage in using TLI over conventional methods of embryo culture and grading [133–135]. Moreover, the high cost of TLI systems, particularly when combined with advanced technologies such as automated assessment algorithms aimed to improve embryo viability assessment, represents an additional constraint. Given the limited cost–benefit ratio and lack of consistent evidence supporting improved outcomes with TLI, there is a continued need for more accurate, cost-effective, and non-invasive alternatives to assess embryo viability.

### Artificial intelligence

Artificial intelligence (AI) has become an important focus in reproductive biology, driven by the growing availability of clinical data, including genomic, proteomic, metabolic, and TLI datasets suitable for meta-analyses and retrospective studies [136]. In general, AI refers to algorithms that use external data to generate analytical and/or predictive functions. While AI encompasses a broad range of computational approaches, the focus of this review will specifically encompass ML as its primary application [136].

To effectively operate an ML algorithm, input data such as patient demographics, medical history, or static embryo images are typically divided into two groups, with approximately 80% of the data used as the “training set” [136–138]. This portion is used to “train” the algorithm to perform calculations, identify correlations, and detect patterns to generate predictions or outputs [138]. The remaining 20% of the data is used as the “test” or “validation set,” allowing researchers to evaluate the algorithm’s predictive accuracy (Figure 3; [136–138]). There are several subsets of ML designed for different types of problems [139]. Supervised learning involves training models on labeled datasets, where the correct outputs are provided, enabling the algorithm to learn mappings from inputs to outputs. In contrast, unsupervised learning works with unlabeled data and focuses on discovering underlying patterns, structures, or groupings within the data. Another major subset is deep learning (DL), which leverages multi-layered neural networks to automatically learn complex representations from large amounts of data, often surpassing traditional ML approaches. In ART, however, DL is most applied to contextualize, analyze, and extract key features from individual or sequential images [138]. Over the past 5 years, there have been significant advances in the use and accuracy of ML models for assessing embryo viability.

**Figure 3:**
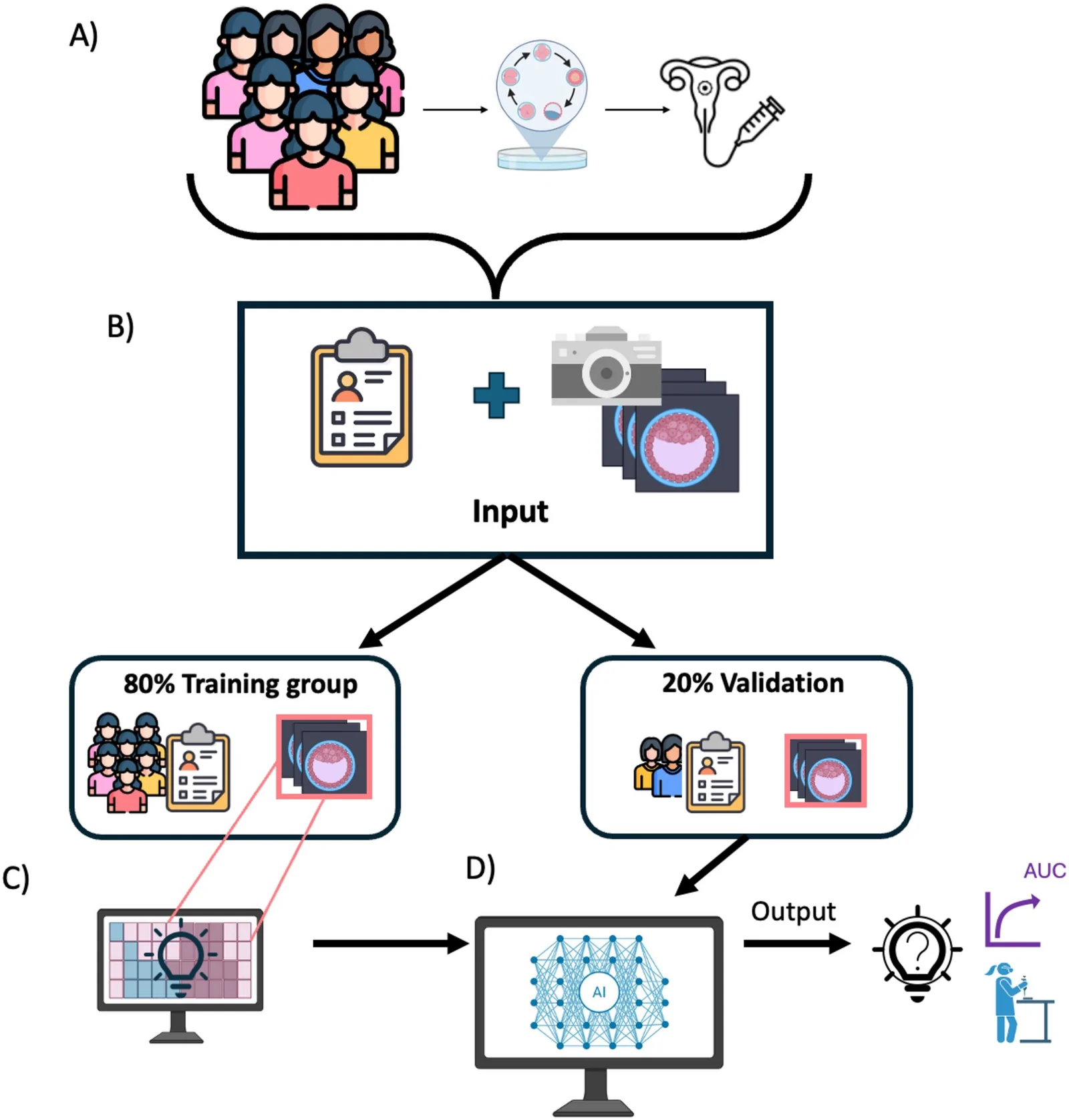
General machine learning methodology for embryo quality evaluation. (A) Patient data collected from individuals who underwent assisted reproductive technologies (ART) procedure; (B) input data, including patient data and corresponding embryo images, are split into two groups: 80% in the training and 20% in the validation group; (C) the algorithm learns to recognize patterns from the input training data; (D) results from the training group is then evaluated by the validation group. Subsequently, output accuracy and precision are determined based on AUC and/or compared to existing methodologies and their golden standards (e.g. embryologist evaluation).

#### Current AI approaches and their limitations

Currently, AI-driven decision support systems (DSSs) are implemented in a limited number of clinics across North America to predict embryo viability through automated morphological grading [140–143]. Morphological grading remains highly subjective and variable, even among experienced embryologists [40, 143]. A review by Salih et al. [143] reported that embryologists are only 51% accurate when estimating pregnancy rates following embryo transfer. By contrast, AI-driven DSSs can consolidate large datasets and identify subtle patterns, thereby enhancing diagnostic efficiency [144]. Subsequently, AI-based systems have the potential to standardize, streamline, and optimize embryo grading, thereby improving both intra- and inter-observer accuracy.

A study by Chavez-Badiola [145] employed the ML algorithm ERICA, using blastocyst images and PGT-A data to predict ploidy status and compared the results with those obtained by experienced embryologists. The algorithm was significantly more accurate than embryologists at ranking euploid embryos, achieving 70% accuracy. As a result, several research teams have been working to develop ML algorithms that integrate TLI data to holistically assess morphological changes during in vitro development [41, 140, 146]. Meseguer et al. [147] used the EmbryoViewer TLI software to establish an optimal timing of cell divisions (cleavage timeframe), identifying these morphokinetic patterns as indicators of embryo developmental potential. Similarly, Kragh et al. [148] demonstrated that combining TLI with an algorithm improved embryo grading and quality assessment compared to those achieved by embryologists.

Moreover, TLI data in ML has been increasingly applied to improve embryo quality assessment by analyzing the algorithm’s ability to extract complex visual data to correctly segregate and classify embryos via their transfer outcome. Bernsten et al. [141] created a DL algorithm, iDAScore v1.0, which analyzed TLI sequences of 115 832 embryos, the largest dataset of its kind to date. Among these, 14 644 embryos had known implantation data (KID), confirmed by the detection of a fetal heartbeat (FH). The researchers evaluated algorithmic performance at segregating embryos (positive or negative FH) using the receiver operating characteristic (ROC), measured by the area under the curve (AUC). The AUC–ROC provides a cumulative score between 0 and 1, which represent how effective an algorithm is at distinguishing true positives from false positives; a higher value denotes greater accuracy at classifying embryos based on image features. When applied to multiple KID embryos (*n* = 1094), iDAScore v1.0 achieved an AUC–ROC of 0.67. Furthermore, another study by Barnes et al. [144] evaluated the ML algorithm STORK-A at distinguishing embryos based on chromosomal status; complex aneuploidy (more than one abnormality), single aneuploidy, and euploidy. A total of 10 378 embryos were biopsied on day 5 and graded on days 5 and 6, with static images captured 110 h post-ICSI. The algorithm incorporated image data, patient age, morphokinetic parameters, and blastocyst grades. STORK-A detected aneuploidy and euploidy with 69.3% accuracy (AUC–ROC: 0.761), and distinguished complex aneuploidy from single aneuploidy/euploidy with 74.0% accuracy (AUC–ROC: 0.760). Comparable outcomes were reported by Ortiz et al. [149] (AUC–ROC: 0.78 for detecting aneuploidy and mosaicism) and Diakiw et al. [150] (AUC–ROC: 0.68 for detecting euploidy).

Deep learning has also been instrumental in enabling automated segmental image analysis (SIA) to evaluate embryo viability [141]. SIA divides an image into multiple regions based on pixel distribution, allowing algorithms to associate quantitative values with specific visual image features [151, 152]. While computational image analysis is well established in fields such as oncology and neuroscience, its application in embryology is still limited. Filho et al. [152] developed an algorithm that used SIA to analyze specific embryo regions, including the TE, ICM, and zona pellucida. Phototextural characteristics such as tone, texture, and complexity were processed through a support vector machine. However, accuracy was low for embryos of poor morphology (<53% for grade B), likely due to the similarity in pixel intensity and texture between the TE and ICM. VerMilyea et al. [140] applied a similar approach with the Life Whisperer AI system, which used 8887 static two-dimensional images of day 5 embryos from 11 IVF clinics to predict viability based on the presence of an FH. The algorithm achieved 64.3% accuracy, and in a blinded test of 801 embryo images, it outperformed embryologists (59.2% vs. 47.4% accuracy). Chen et al. [151] formulated the algorithm Embryo-Net, which employs spatial modeling to more accurately distinguish pixel differences between the ICM and TE, outperforming earlier algorithms. However, all SIA models inherently assume that the ICM is “morphologically regular” and located centrally, an assumption not applicable to embryos of with irregular shapes or at different developmental stages [151]. For instance, ovoid embryos have been shown to exhibit implantation rates comparable to normal embryos when assisted hatching is applied [153]. Therefore, segmentation of embryo structures in embryos of diverse dimensions remains a significant limitation to the clinical integration of SIA-based AI systems, as embryo morphology is often highly variable.

Although studies demonstrate the potential of AI to reduce subjectivity and increase accuracy in embryo viability assessment [140], several additional limitations restrict its clinical reliability. Chief among these include ethical, legal, and data privacy concerns. The accuracy of ML algorithms is highly dependent on the quantity and quality of input data, which often include sensitive patient information such as ethnicity, family history, medical conditions, and body mass index [144, 145]. Studies show that integrating clinical data alongside morphokinetic and imaging features improves model accuracy [144, 145]. De Gheselle et al. [154] found that algorithms based solely on morphokinetic features achieved 56% accuracy, while combining morphokinetic and embryonic developmental features increased accuracy to 67%. When patient demographic and clinical data were included, accuracy further improved to 72%. Despite these advantages, privacy and data security remain significant barriers. A survey by Yang et al. [155] revealed that many healthcare professionals express concerns about AI and patient data protection, while Shoham et al. [156] reported that 11.1% of embryologists and physicians cited fear of privacy violations. These concerns are exacerbated by the “black box” nature of many AI models, including those developed by Bernsten et al. [141], which cannot be independently verified or audited. Additionally, while AI tools are designed to assist clinical staff, their increasing presence has shifted training dynamics. Kim et al. [157] reported that junior embryologists rely more heavily on AI for morphological assessment than senior embryologists, raising concerns about the erosion of technical and observational skills. Although ML improves accuracy for both groups of embryologists, this dependence may hinder skill development among trainees. Another critical issue is overfitting, which occurs when an algorithm memorizes training data rather than learning generalizable patterns, limiting its applicability to new datasets [158]. This issue is especially common in studies with small sample sizes, leading to an inflated perception of algorithmic accuracy.

Given these limitations, AI cannot yet be considered a fully reliable, non-invasive tool for assessing embryo viability in clinical settings. AI is the most promising development in improving the efficacy of ART via embryo viability assessment. Future development should focus on quantitative, data-independent algorithms that minimize reliance on sensitive patient information while maintaining predictive accuracy.

## Ultra-weak photon emission or “biophotons”

Mammalian oocytes and early preimplantation embryos emit extremely weak spontaneous light (<100 photons cm^−2^ s^−1^), known as ultra-weak photon emission (UPE). This emission reflects underlying metabolic and oxidative processes and, in principle, may serve as a non-invasive indicator of embryo viability. Recent reviews (e.g. Mould et al. [159]) have outlined UPE detection methods, proposed biochemical sources, including oxidative and other metabolic reactions, and discussed potential biomedical applications. That publication also highlights major technical advances (e.g. low-noise imaging sensors and photomultiplier tubes) that now make UPE measurements in embryos increasingly feasible. Studies by Bodis et al. [160] and Berke et al. [161] demonstrated detectable UPE signals from mouse embryos. Using specialized imaging pipelines and advanced signal-processing algorithms, these studies reported differences in photon emission intensity between viable and degenerated embryos, suggesting that UPE may provide a functional biomarker of embryo developmental competence. While UPE has been investigated in various human tissues including whole-body measurements [162], there are currently no published studies measuring UPE directly from human preimplantation embryos.

## r-Algo

r-Algo 2.0 is a proprietary image-analysis software developed at the University of Guelph to improve the repeatability, accuracy, and precision of image analysis through discriminative segmentation analyses of digital grayscale image texture [163–165]. Image texture refers to how the intensity or brightness of an image represents the structural composition of an object [163, 165]. r-Algo 2.0 quantifies image features using bitmap analysis, which is the transformation of a digital, grayscale image into a numerical grid for quantitative analysis. A bitmap “translates” an image into a numerical format by assigning each individual pixel a specific grayscale intensity value on the 8-bit scale from 0 (absolute black) to 255 (absolute white; Figure 4) [165]. This pixel-based structure enables r-Algo 2.0 to perform highly granular analyses of image phototextural attributes by assessing the spatial distribution and frequency of pixel intensities within user-defined regions of interest (ROIs) [165].

**Figure 4:**
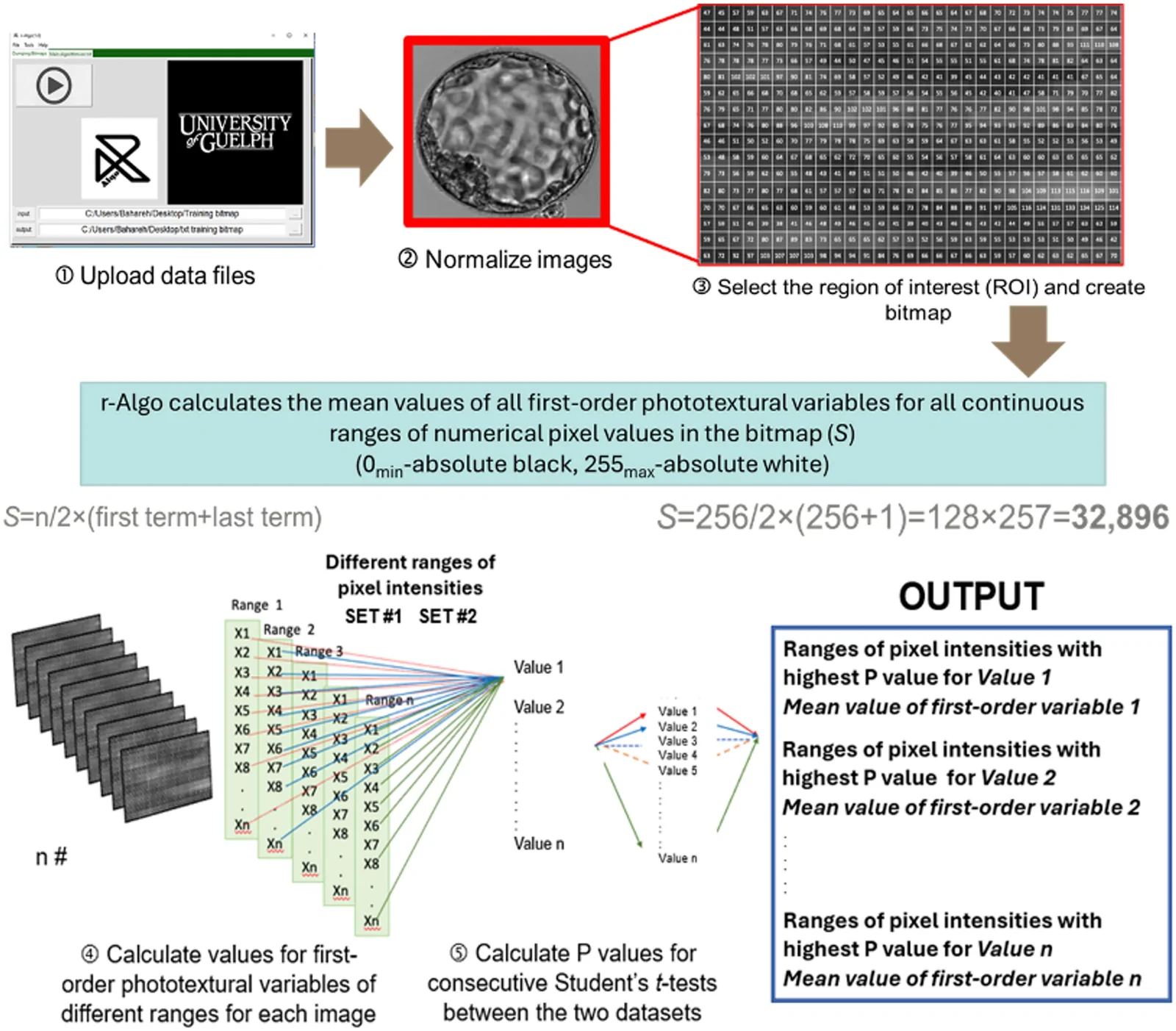
A flow chart demonstrating the analytical steps of the r-Algo 2.0 software, a quantitative Python algorithm that performs segmental analysis of bitmapped images, for the detection of quantitative differences between two datasets or input sources. *S* = sum of the series or the number of all continuous pixel-intensity ranges in the bitmap; *n* = 256 (0–255).

Unlike traditional image-analysis approaches, which rely heavily on operator interpretation, r-Algo 2.0 applies automated pattern-recognition algorithms to extract quantitative metrics that reflect underlying image microarchitecture [165]. r-Algo 2.0 calculates the mean values of first-order textural variables for all continuous ranges of numerical pixel values in the bitmap, allowing for a total of 32 896 combinations of pixel-intensity ranges being analyzed [164, 165]. First-order textural characteristics are basic statistical measures derived directly from the intensity histogram of the ROI including mean pixel intensity (MPI), heterogeneity (MPH), and pixel distribution concentration (MPC) [164, 165]. MPI is the average brightness of all pixels within the ROI, MPH is the standard deviation of MPI and represents variability in pixel brightness, and MPC is the proportion of pixels falling within a specific echointensity region relative to the total number of pixels in the ROI, providing insight into the prevalence of specific grayscale zones [165]. Previous literature indicates that r-Algo 2.0 is adaptable across a wide range of biological ultrasound, histological, and radiological images, making it a versatile tool for cell and tissue characterization, including applications in reproductive biology [163, 165].

Most recently, r-Algo 2.0 was applied to investigate whether grayscale phototexture of the cytoplasm of ovine presumptive zygotes could predict their developmental potential during IVC [164]. Using high-resolution images obtained from TLI incubators, researchers analyzed microphotographs of presumptive zygotes shortly after fertilization and extracted their phototextural features. Findings revealed that within r-Algo identified pixel-intensity ranges, ovine embryos that successfully developed into blastocysts exhibited significantly higher MPI and MPC values but had lower MPH compared to those that arrested prior to blastulation [164]. The accuracy of identifying future arresting embryos from phototextural attributes of zygotic cytoplasm was 86% for MPI, 83% for MPH, and 85% for MPC [164]. These results suggest that specific phototextural signatures revealed by algorithmic image analysis, likely reflecting cytoplasmic uniformity or subcellular organization, are indicative of zygotic developmental competence.

### r-Algo 2.0 as a promising non-invasive approach to PGT-A

Based largely on the work described by Bartlewski et al. [164], it was proposed that the r-Algo 2.0 system could enable detection of embryo ploidy status based on phototextural differences between aneuploid and euploid day-5 human blastocysts. A preliminary study was conducted at the University of Guelph [166] in partnership with the Institute for the Diagnosis and Treatment of Infertility—KrakOvi (Cracow, Poland), following informed consent from 50 female patients undergoing infertility treatment for both maternal (age: 29–42) and paternal factors. After ICSI, digital microphotographs of day-5 blastocysts (78 euploid, 51 aneuploid) were obtained immediately prior to PGT-A. Microphotographs were cropped, converted to 8-bit grayscale using ImagePro Plus 7.0 analytical software (Media Cybernetics Inc., Rockville, MD, USA), and normalized using r-Algo 2.0. Bitmaps of normalized microphotographs were generated by r-Algo 2.0 to identify pixel-intensity ranges (PIRs) with first-order phototextural characteristics that differed between aneuploid and euploid blastocysts. Significant differences were detected across the following short PIRs: MPI (32–42), MPH (196–198), and MPC (164–165). All first-order phototextural characteristics were higher in euploid than aneuploid embryos (Figure 5).

**Figure 5:**
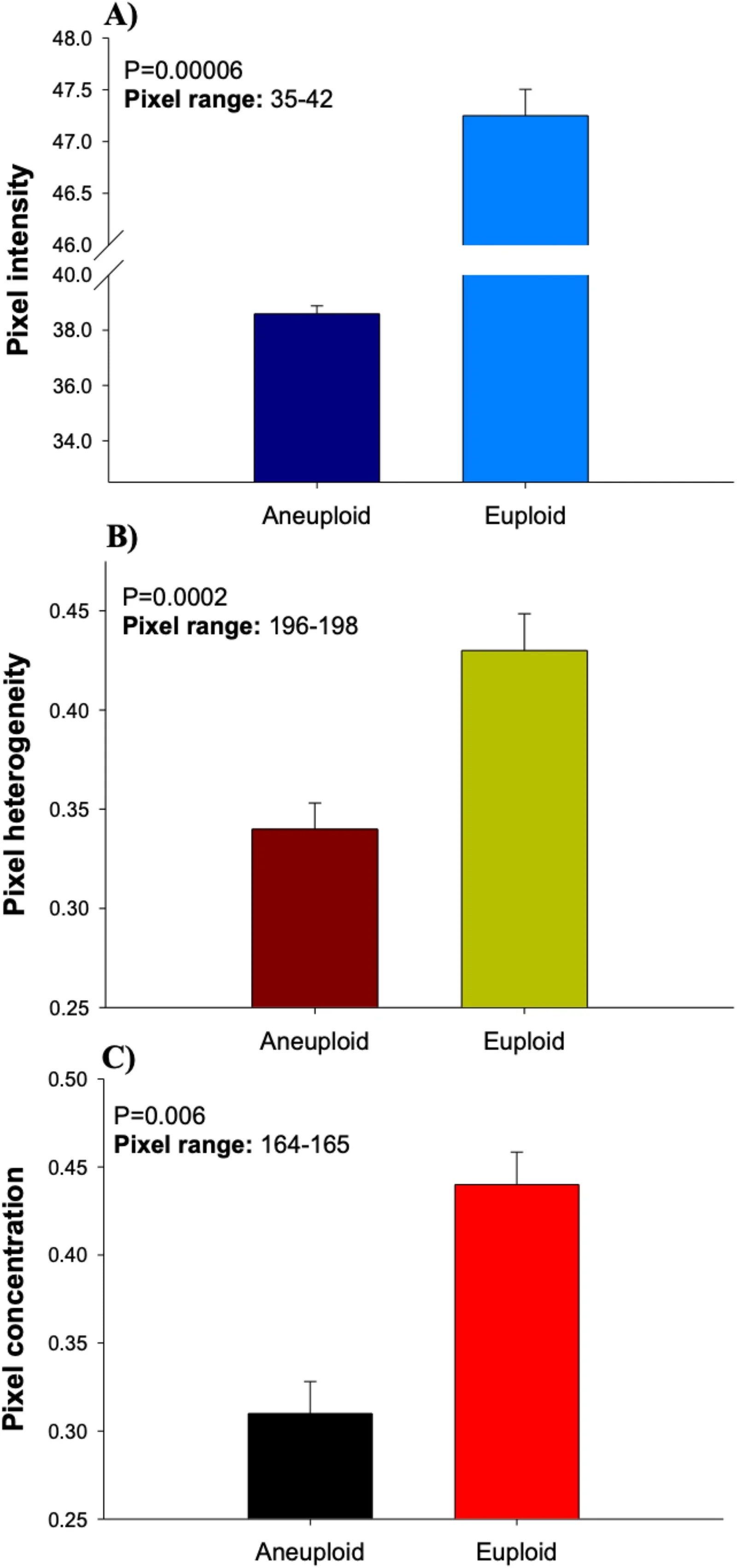
Bar graphs (means ± SEMs) indicating statistically significant differences in first-order phototextural characteristics between aneuploid and euploid ICSI-derived blastocysts within algorithmically identified specific pixel-intensity ranges [166].

Phototextural differences between aneuploid and euploid embryos could be attributed to morphological differences, such as fewer or more loosely packed cells within the ICM and TE, which are features commonly associated with aneuploidy [167]. As established by Khajehoseini et al. [168], aneuploid embryos exhibit increased concentrations of BF-cfDNA, which may impact the fluidity of the blastocoel cavity. Additionally, aneuploid blastocysts may experience cytoplasmic dysmorphism, similar to that observed in oocytes and zygotes, including smooth endoplasmic reticulum aggregation or abnormal distribution of mitochondria [169, 170]. The narrow ranges of pixel-intensity values for the three phototextural parameters (MPI-8, MPH-3, and MPC-3) suggest that microscopically detectable differences in cytomorphology between euploid and aneuploid embryos are very subtle or may be confined to relatively small regions or compartments within the blastocyst. Overall, statistically significant results support the use of quantitative, algorithmic phototextural analysis as a promising non-invasive method for ploidy detection.

## Concluding remarks

Current methods of embryo assessment are invasive, time-consuming, and/or costly (e.g. PGT-A, blastocoel fluid assessment) and remain subjective (e.g. microscopic grading). Based on the available literature, machine learning (ML) represents a more promising option for the non-invasive assessment of embryo viability compared to spent culture media (SCM) and blastocoel fluid (BF) analysis. Both SCM and BF approaches fail to predict ploidy status with the same efficiency as traditional PGT-A [171, 172]. Although SCM generally demonstrates a higher amplification rate than BF, contamination from non-embryonic sources remains a major limitation [97, 172]. Evidence indicates that ML algorithms are steadily improving in accuracy and can predict ploidy status more effectively than experienced embryologists [172]. While it is unlikely that ML algorithms can fully replace methods such as PGT-A, continued optimization of algorithmic application, such as addressing issues such as overfitting, could potentially allow ML algorithms to serve as a complementary and cost-effective method for measuring embryo viability. To promote effective clinical integration, embryologists should contribute to the development of comprehensive, standardized databases incorporating embryo images, metabolomic profiles, and other relevant parameters, thereby minimizing sample-size bias and improving algorithm performance.

We demonstrated that r-Algo 2.0 is a promising, non-invasive tool for assessing embryo ploidy status. Because r-Algo 2.0 performs quantitative segmental analysis on image bitmaps, it fills a major gap identified in the current literature. The r-Algo software can be considered a “glass-box” algorithm, as both its data interpretation process and output generation are clearly defined for the user. The selection of first-order phototextural features and the calculation of indicative pixel-intensity ranges are transparent and easily interpretable. Despite its simplicity and the advantages outlined above, further validation is required to confirm its efficacy. Future development of r-Algo 2.0 will focus on improving image acquisition to minimize background artifacts, optimizing image conditions (e.g. brightness and camera quality), and refining analysis of specific regions of interest (ROI; e.g. the trophectoderm (TE) and inner cell mass (ICM) compartments) to enhance the algorithm’s predictive accuracy and diagnostic sensitivity as an independent diagnostic tool or in comprehensive embryo scoring schemes.

## Supplementary Material

Supplementary_material_ioag147

## References

[1] “1 in 6 people globally affected by infertility: WHO,” World Health Organization, Geneva, Switzerland. Accessed: Nov. 30, 2024. [Online]. Available: https://www.who.int/news/item/04-04-2023-1-in-6-people-globally-affected-by-infertility

[2] SART . SART National Summary Report. SART 2024. [Online]. Available: https://www.sartcorsonline.com/CSR/PublicSnapshotReport?ClinicPKID=&reportingYear=2024&fromDisclaimer=true.

[3] Greil AL . Infertility and psychological distress: a critical review of the literature. Soc Sci Med 1997; 45:1679–1704. 10.1016/S0277-9536(97)00102-0. 9428088

[4] Sharma A, Shrivastava D. Psychological problems related to infertility. Cureus 2022; 10:30320. 10.7759/cureus.30320. PMC9661871 36407201

[5] Practice Committee of the American Society for Reproductive Medicine and the Practice Committee for the Society for Assisted Reproductive Technologies . Guidance on the limits to the number of embryos to transfer: a committee opinion. Fertil Steril 2021; 116:651–654. 10.1016/j.fertnstert.2021.06.050. 34330423

[6] Reimundo P, Gutiérrez Romero JM, Rodríguez Pérez T, Veiga E. Single-embryo transfer: a key strategy to reduce the risk for multiple pregnancy in assisted human reproduction. Adv Lab Med 2021; 2:179–198. 10.1515/almed-2021-0013. PMC10197809 37363329

[7] Davis OS, Favetta LA, Deniz S, Faghih M, Amin S, Karnis M, Neal MS. Potential costs and benefits of incorporating PGT-A across age groups: a Canadian clinic perspective. J Obstet Gynaecol Can 2024; 46:102361. 10.1016/j.jogc.2024.102361. 38272217

[8] Bashiri Z, Afzali A, Koruji M, Torkashvand H, Ghorbanlou M, Sheibak N, Zandieh Z, Amjadi F. Advanced strategies for single embryo selection in assisted human reproduction: a review of clinical practice and research methods. Clin Exp Reprod Med 2025; 52:8–29. 10.5653/cerm.2023.06478. PMC11900676 38853126

[9] Somigliana E, Busnelli A, Paffoni A, Vigano P, Riccaboni A, Rubio C, Capalbo A. Cost-effectiveness of preimplantation genetic testing for aneuploidies. Fertil Steril 2019; 111:1169–1176. 10.1016/j.fertnstert.2019.01.025. 30777289

[10] Neal SA, Franasiak JM, Forman EJ, Werner MD, Morin SJ, Tao X, Treff NR, Scott RT Jr. High relative deoxyribonucleic acid content of trophectoderm biopsy adversely affects pregnancy outcomes. Fertil Steril 2017; 107:731–736.e1. 10.1016/j.fertnstert.2016.11.013. 27939761

[11] Paulson RJ . Hidden in plain sight: the overstated benefits and underestimated losses of potential implantations associated with advertised PGT-A success rates. Hum Reprod 2020; 35:490–493. 10.1093/humrep/dez280. 32100030

[12] Rolfes V, Bittner U, Gerhards H, Krüssel JS, Fehm T, Ranisch R, Fangerau H. Artificial intelligence in reproductive medicine – an ethical perspective. Geburtshilfe Frauenheilkd 2023; 83:106–115. 10.1055/a-1866-2792. PMC9833891 36643877

[13] Tesarik J, Greco E. The probability of abnormal preimplantation development can be predicted by a single static observation on pronuclear stage morphology. Hum Reprod 1999; 14:1318–1323. 10.1093/humrep/14.5.1318. 10325285

[14] Scott L . Pronuclear scoring as a predictor of embryo development. Reprod Biomed Online 2003; 6:201–214. 10.1016/S1472-6483(10)61711-7. 12676001

[15] Stigliani S, Massarotti C, Bovis F, Casciano I, Sozzi F, Remorgida V, Cagnacci A, Anserini P, Scaruffi P. Pronuclear score improves prediction of embryo implantation success in ICSI cycles. BMC Pregnancy Childbirth 2021; 21:361. 10.1186/s12884-021-03820-7. PMC8097973 33952184

[16] Alpha Scientists in Reproductive Medicine and ESHRE Special Interest Group of Embryology, et al. The Istanbul consensus workshop on embryo assessment: proceedings of an expert meeting. Hum Reprod 2011; 26:1270–1283. 10.1093/humrep/der037. 21502182

[17] Nagy ZP, Dozortsev D, Diamond M, Rienzi L, Ubaldi F, Abdelmassih R, Greco E. Pronuclear morphology evaluation with subsequent evaluation of embryo morphology significantly increases implantation rates. Fertil Steril 2003; 80:67–74. 10.1016/S0015-0282(03)00569-7. 12849803

[18] Tesarik J, Junca AM, Hazout A, Aubriot FX, Nathan C, Cohen-Bacrie P, Dumont-Hassan M. Embryos with high implantation potential after intracytoplasmic sperm injection can be recognized by a simple, non-invasive examination of pronuclear morphology. Hum Reprod 2000; 15:1396–1399. 10.1093/humrep/15.6.1396. 10831576

[19] Li D, Parmegiani L, Yang D, Vajta G, Li R. Expert consensus on the morphological evaluation of human cleavage-stage embryos and blastocysts. Chin Med J (Engl) 2023; 136:1009–1011. 10.1097/CM9.0000000000002609. PMC10228470 37185360

[20] Desai NN, Goldstein J, Rowland DY, Goldfarb JM. Morphological evaluation of human embryos and derivation of an embryo quality scoring system specific for day 3 embryos: a preliminary study. Hum Reprod Oxf Engl 2000; 15:2190–2196. 10.1093/humrep/15.10.2190. 11006197

[21] Stensen MH, Tanbo T, Storeng R, Byholm T, Fèdorcsak P. Routine morphological scoring systems in assisted reproduction treatment fail to reflect age-related impairment of oocyte and embryo quality. Reprod Biomed Online 2010; 21:118–125. 10.1016/j.rbmo.2010.03.018. 20452822

[22] Balaban B, Urman B, Alatas C, Mercan R, Aksoy S, Isiklar A. Blastocyst-stage transfer of poor-quality cleavage-stage embryos results in higher implantation rates. Fertil Steril 2001; 75:514–518. 10.1016/S0015-0282(00)01756-8. 11239534

[23] Liu Z, Cai J, Liu L, Ouyang L, Chen J, Yang C, Chen K, Yang X, Ren J, Jiang X. Does cleavage stage morphology increase the discriminatory power of prediction in blastocyst transfer outcome? J Assist Reprod Genet 2024; 41:347–358. 10.1007/s10815-023-02997-4. PMC10894791 38040894

[24] Zilberberg E, Casper R, Meriano J, Barzilay E, Aizer A, Kirshenbaum M, Orvieto R, Haas J. Cleavage vs blastocyst stage embryos: how are they interrelating? Arch Gynecol Obstet 2021; 304:1083–1088. 10.1007/s00404-021-06003-z. 33620556

[25] Kong X, Yang S, Gong F, Lu C, Zhang S, Lu G, Lin G. The relationship between cell number, division behavior and developmental potential of cleavage stage human embryos: a time-lapse study. PloS One 2016; 11:e0153697. 10.1371/journal.pone.0153697. PMC4831697 27077739

[26] Yang S-H, Wu CH, Chen YC, Yang CK, Wu TH, Chen PC, Tsai HD. Effect of morphokinetics and morphological dynamics of cleavage stage on embryo developmental potential: a time-lapse study. Taiwan J Obstet Gynecol 2018; 57:76–82. 10.1016/j.tjog.2017.12.013. 29458909

[27] Bartolacci A, Dal Canto M, Guglielmo MC, Mura L, Brigante C, Mignini Renzini M, Buratini J. Early embryo morphokinetics is a better predictor of post-ICSI live birth than embryo morphology: speed is more important than beauty at the cleavage stage. Zygote 2021; 29:495–502. 10.1017/S0967199421000253. 33910671

[28] Dal Canto M, Bartolacci A, Turchi D, Pignataro D, Lain M, de Ponti E, Brigante C, Mignini Renzini M, Buratini J. Faster fertilization and cleavage kinetics reflect competence to achieve a live birth after intracytoplasmic sperm injection, but this association fades with maternal age. Fertil Steril 2021; 115:665–672. 10.1016/j.fertnstert.2020.06.023. 32888678

[29] Lee C-I, Lee T-H, Huang C-C, Chen H-H, Liu C-H, Lee M-S. Detection of early cleavage embryos improves pregnancy and delivery rates of day 3 embryo transfer during in vitro fertilization. Taiwan J Obstet Gynecol 2016; 55:558–562. 10.1016/j.tjog.2016.06.005. 27590382

[30] The Working Group on the update of the ESHRE/ALPHA Istanbul Consensus, et al. The Istanbul consensus update: a revised ESHRE/ALPHA consensus on oocyte and embryo static and dynamic morphological assessment. Hum Reprod 2025; 40:989–1035. 10.1093/humrep/deaf021. PMC12127515 40288770

[31] Martello C, Andrade G, Frantz G, Pagnoncelli N, Dutra C, Rockenbach M, Donatti L, Frantz N. P-284 Correlation between a new day 4 embryo scoring system and blastocyst development and pregnancy rate. Hum Reprod 2022; 37:deac107.273. 10.1093/humrep/deac107.273.

[32] Fabozzi G, Alteri A, Rega E, Starita MF, Piscitelli C, Giannini P, Colicchia A. Morphological assessment on day 4 and its prognostic power in selecting viable embryos for transfer. Zygote 2016; 24:477–484. 10.1017/S0967199415000404. 26350430

[33] Gardner DK, Schoolcraft W. In vitro culture of human blastocysts. In: Jansen R, Mortimer D (eds.), Towards Reproductive Certainty: Fertility and Genetics Beyond. Sydney, Austrailia: Parthenon Publishing Group; 1999:378–388.

[34] Gardner DK, Lane M, Stevens J, Schlenker T, Schoolcraft WB. Blastocyst score affects implantation and pregnancy outcome: towards a single blastocyst transfer. Fertil Steril 2000; 73:1155–1158. 10.1016/S0015-0282(00)00518-5. 10856474

[35] Alfarawati S, Fragouli E, Colls P, Stevens J, Gutiérrez-Mateo C, Schoolcraft WB, Katz-Jaffe MG, Wells D. The relationship between blastocyst morphology, chromosomal abnormality, and embryo gender. Fertil Steril 2011; 95:520–524. 10.1016/j.fertnstert.2010.04.003. 20537630

[36] Karaki RZ, Samarraie SS, Younis NA, Lahloub TM, Ibrahim MH. Blastocyst culture and transfer: a step toward improved in vitro fertilization outcome. Fertil Steril 2002; 77:114–118. 10.1016/s0015-0282(01)02939-9. 11779600

[37] Kalu E, Thum M-Y, Abdalla H. Reducing multiple pregnancy in assisted reproduction technology: towards a policy of single blastocyst transfer in younger women. BJOG 2008; 115:1143–1150. 10.1111/j.1471-0528.2008.01764.x. 18518874

[38] Styer AK, Wright DL, Wolkovich AM, Veiga C, Toth TL. Single-blastocyst transfer decreases twin gestation without affecting pregnancy outcome. Fertil Steril 2008; 89:1702–1708. 10.1016/j.fertnstert.2007.05.036. 17644095

[39] Wilson M, Hartke K, Kiehl M, Rodgers J, Brabec C, Lyles R. Integration of blastocyst transfer for all patients. Fertil Steril 2002; 77:693–696. 10.1016/s0015-0282(01)03235-6. 11937117

[40] Storr A, Venetis CA, Cooke S, Kilani S, Ledger W. Inter-observer and intra-observer agreement between embryologists during selection of a single day 5 embryo for transfer: a multicenter study. Hum Reprod 2017; 32:307–314. 10.1093/humrep/dew330. 28031323

[41] Boucret L, Tramon L, Saulnier P, Ferré-L’Hôtellier V, Bouet P-E, May-Panloup P. Change in the strategy of embryo selection with time-lapse system implementation—impact on clinical pregnancy rates. J Clin Med 2021; 10:4111. 10.3390/jcm10184111. PMC8471151 34575222

[42] Li N, Guan Y, Ren B, Zhang Y, du Y, Kong H, Zhang Y, Lou H. Effect of blastocyst morphology and developmental rate on euploidy and live birth rates in preimplantation genetic testing for aneuploidy cycles with single-embryo transfer. Front Endocrinol 2022; 13:858042. 10.3389/fendo.2022.858042. PMC9044033 35498424

[43] Santamonkunrot P, Samutchinda S, Niransuk P, Satirapod C, Sukprasert M. The association between embryo development and chromosomal results from PGT-A in women of advanced age: a prospective cohort study. J Clin Med 2024; 13:626. 10.3390/jcm13020626. PMC10816670 38276130

[44] Cornelisse S, Zagers M, Kostova E, Fleischer K, Van Wely M, Mastenbroek S. Preimplantation genetic testing for aneuploidies (abnormal number of chromosomes) in in vitro fertilisation. Cochrane Database Syst Rev 2020; 9:CD005291. 10.1002/14651858.CD005291.pub3. PMC8094272 32898291

[45] De Rycke M, Berckmoes V. Preimplantation genetic testing for monogenic disorders. Genes 2020; 11:871. 10.3390/genes11080871. PMC7463885 32752000

[46] Handyside AH, Kontogianni EH, Hardy K, Winston RML. Pregnancies from biopsied human preimplantation embryos sexed by Y-specific DNA amplification. Nature 1990; 344:768–770. 10.1038/344768a0. 2330030

[47] Gibbons WE, Gitlin SA, Lanzendorf SE, Kaufmann RA, Slotnick RN, Hodgen GD. Preimplantation genetic diagnosis for Tay-Sachs disease: successful pregnancy after pre-embryo biopsy and gene amplification by polymerase chain reaction. Fertil Steril 1995; 63:723–728. 10.1016/S0015-0282(16)57472-X. 7890054

[48] Solovova OA, Chernykh VB. Genetics of oocyte maturation defects and early embryo development arrest. Genes 2022; 13:1920. 10.3390/genes13111920. PMC9689903 36360157

[49] Vlachadis N, et al. Incidence and types of chromosomal abnormalities in first trimester spontaneous miscarriages: a Greek single-center prospective study. Maedica 2023; 18:35–41. 10.26574/maedica.2023.18.1.35. PMC10231156 37266460

[50] Robertson SA, Richards RI. Single-cell sequencing shows mosaic aneuploidy in most human embryos. J Clin Invest 2024; 134:e179134. 10.1172/JCI179134. PMC10940079 38488008

[51] Volodyaev I, Mazunin I, Semenova M, Korchivaya E, Ivanova A. Chromosomal abnormalities in embryo development: what do we really know about them and what have we made up? Reprod Biomed Online 2024; 48:104031. 10.1016/j.rbmo.2024.104031.

[52] Capalbo A, Poli M, Rienzi L, Girardi L, Patassini C, Fabiani M, Cimadomo D, Benini F, Farcomeni A, Cuzzi J, Rubio C, Albani E, et al. Mosaic human preimplantation embryos and their developmental potential in a prospective, non-selection clinical trial. Am J Hum Genet 2021; 108:2238–2247. 10.1016/j.ajhg.2021.11.002. PMC8715143 34798051

[53] Popovic M, Dhaenens L, Boel A, Menten B, Heindryckx B. Erratum. Chromosomal mosaicism in human blastocysts: the ultimate diagnostic dilemma. Hum Reprod Update 2020; 26:450–451. 10.1093/humupd/dmaa015. 32298446

[54] ESHRE Working Group on Chromosomal Mosaicism, et al. ESHRE survey results and good practice recommendations on managing chromosomal mosaicism. Hum Reprod Open 2022; 2022:hoac044. 10.1093/hropen/hoac044. PMC9637425 36349144

[55] Spinella F, Fiorentino F, Biricik A, Bono S, Ruberti A, Cotroneo E, Baldi M, Cursio E, Minasi MG, Greco E. Extent of chromosomal mosaicism influences the clinical outcome of in vitro fertilization treatments. Fertil Steril 2018; 109:77–83. 10.1016/j.fertnstert.2017.09.025. 29191449

[56] Lee C-I, Cheng EH, Lee MS, Lin PY, Chen YC, Chen CH, Huang LS, Huang CC, Lee TH. Healthy live births from transfer of low-mosaicism embryos after preimplantation genetic testing for aneuploidy. J Assist Reprod Genet 2020; 37:2305–2313. 10.1007/s10815-020-01876-6. PMC7492347 32623662

[57] Munné S, Spinella F, Grifo J, Zhang J, Beltran MP, Fragouli E, Fiorentino F. Clinical outcomes after the transfer of blastocysts characterized as mosaic by high resolution next generation sequencing—further insights. Eur J Med Genet 2020; 63:103741. 10.1016/j.ejmg.2019.103741. 31445143

[58] Das P, et al. New insights into elucidating the pregnancy outcome of low mosaic embryo transfer. Reprod Biomed Online 2024; 48:104021. 10.1016/j.rbmo.2024.104021.

[59] Bhatt SJ, Marchetto NM, Roy J, Morelli SS, McGovern PG. Pregnancy outcomes following *in vitro* fertilization frozen embryo transfer (IVF-FET) with or without preimplantation genetic testing for aneuploidy (PGT-A) in women with recurrent pregnancy loss (RPL): a SART-CORS study. Hum Reprod 2021; 36:2339–2344. 10.1093/humrep/deab117. 34027546

[60] Harris BS, Acharya KS, Unnithan S, Neal SA, Mebane S, Truong T, Muasher SJ. Success rates with preimplantation genetic testing for aneuploidy in good prognosis patients are dependent on age. Fertil Steril 2025; 123:428–438. 10.1016/j.fertnstert.2024.09.043. 39349118

[61] Wirleitner B, Hrubá M, Schuff M, Hradecký L, Stecher A, Damko A, Stadler J, Spitzer D, Obkircher M, Murtinger M. Embryo drop-out rates in preimplantation genetic testing for aneuploidy (PGT-A): a retrospective data analysis from the DoLoRes study. J Assist Reprod Genet 2024; 41:193–203. 10.1007/s10815-023-02976-9. PMC10789689 37878220

[62] Ginoza MEC, Isasi R. Regulating preimplantation genetic testing across the world: a comparison of international policy and ethical perspectives. Cold Spring Harb Perspect Med 2020; 10:a036681. 10.1101/cshperspect.a036681. PMC7197420 31506325

[63] Wyroba J, Kochan J, Barszcz M, Mirocki G, Kordowitzki P. Reproductive aging, preimplantation genetic testing for aneuploidy, and the diameter of blastocysts: does size matter? Aging 2025; 17:630–642. 10.18632/aging.206215. PMC11984427 40050021

[64] Cross-border reproductive care: an Ethics Committee opinion . Fertil Steril 2022; 117:954–962. 10.1016/j.fertnstert.2022.01.012. 35216836

[65] Arav A, Aroyo A, Yavin S, Roth Z. Prediction of embryonic developmental competence by time-lapse observation and ‘shortest-half’ analysis. Reprod Biomed Online 2008; 17:669–675. 10.1016/S1472-6483(10)60314-8. 18983751

[66] Lundin K, Park H. Time-lapse technology for embryo culture and selection. Ups J Med Sci 2020; 125:77–84. 10.1080/03009734.2020.1728444. PMC7720962 32096675

[67] Belandres D, Shamonki M, Arrach N. Current status of spent embryo media research for preimplantation genetic testing. J Assist Reprod Genet 2019; 36:819–826. 10.1007/s10815-019-01437-6. PMC6541676 30895497

[68] Kobayashi M, Kobayashi J, Shirasuna K, Iwata H. Abundance of cell-free mitochondrial DNA in spent culture medium associated with morphokinetics and blastocyst collapse of expanded blastocysts. Reprod Med Biol 2020; 19:404–414. 10.1002/rmb2.12344. PMC7542021 33071643

[69] Chronopoulou E, Harper JC. IVF culture media: past, present and future. Hum Reprod Update 2015; 21:39–55. 10.1093/humupd/dmu040. 25035437

[70] Sunde A, Brison D, Dumoulin J, Harper J, Lundin K, Magli MC, van den Abbeel E, Veiga A. Time to take human embryo culture seriously. Hum Reprod 2016; 31:2174–2182. 10.1093/humrep/dew157. 27554442

[71] Gott AL, Hardy K, Winston RML, Leese HJ. Non-invasive measurement of pyruvate and glucose uptake and lactate production by single human preimplantation embryos. Hum Reprod 1990; 5:104–108. 10.1093/oxfordjournals.humrep.a137028. 2324239

[72] Hardy K, Hooper MAK, Handyside AH, Rutherford AJ, Winston RML, Leese HJ. Non-invasive measurement of glucose and pyruvate uptake by individual human oocytes and preimplantation embryos. Hum Reprod 1989; 4:188–191. 10.1093/oxfordjournals.humrep.a136869. 2918073

[73] Leese HJ, McKeegan PJ, Sturmey RG. Amino acids and the early mammalian embryo: origin, fate, function and life-long legacy. Int J Environ Res Public Health 2021; 18:9874. 10.3390/ijerph18189874. PMC8467587 34574797

[74] Bentley R . Secondary metabolite biosynthesis: the first century. Crit Rev Biotechnol 1999; 19:1–40. 10.1080/0738-859991229189. 10230052

[75] Renard JP, Philippon A, Menezo Y. In-vitro uptake of glucose by bovine blastocysts. *J Reprod Fertil* 1980; 58:161–164. 10.1530/jrf.0.0580161. 7359473

[76] Gardner DK, Wale PL, Collins R, Lane M. Glucose consumption of single post-compaction human embryos is predictive of embryo sex and live birth outcome. Hum Reprod 2011; 26:1981–1986. 10.1093/humrep/der143. 21572086

[77] Gardner DK, Wale PL. Analysis of metabolism to select viable human embryos for transfer. Fertil Steril 2013; 99:1062–1072. 10.1016/j.fertnstert.2012.12.004. 23312219

[78] Picton HM, Elder K, Houghton FD, Hawkhead JA, Rutherford AJ, Hogg JE, Leese HJ, Harris SE. Association between amino acid turnover and chromosome aneuploidy during human preimplantation embryo development in vitro. Mol Hum Reprod 2010; 16:557–569. 10.1093/molehr/gaq040. PMC2907220 20571076

[79] Miao S-B, Feng YR, Wang XD, Lian KQ, Meng FY, Song G, Yuan JC, Geng CP, Wu XH. Glutamine as a potential noninvasive biomarker for human embryo selection. Reprod Sci 2022; 29:1721–1729. 10.1007/s43032-021-00812-y. PMC9110480 35075614

[80] Sánchez-Ribas I, Riqueros M, Vime P, Puchades-Carrasco L, Jönsson T, Pineda-Lucena A, Ballesteros A, Domínguez F, Simón C. Differential metabolic profiling of non–pure trisomy 21 human preimplantation embryos. Fertil Steril 2012; 98:1157–1164.e2. 10.1016/j.fertnstert.2012.07.1145. 22959456

[81] Pinto S, Guerra-Carvalho B, Crisóstomo L, Rocha A, Barros A, Alves MG, Oliveira PF. Metabolomics integration in assisted reproductive technologies for enhanced embryo selection beyond morphokinetic analysis. Int J Mol Sci 2024; 25:491. 10.3390/ijms25010491. PMC10778973 38203668

[82] Eldarov C, Gamisonia A, Chagovets V, Ibragimova L, Yarigina S, Smolnikova V, Kalinina E, Makarova N, Zgoda V, Sukhikh G, Bobrov M. LC-MS analysis revealed the significantly different metabolic profiles in spent culture media of human embryos with distinct morphology, karyotype and implantation outcomes. Int J Mol Sci 2022; 23:2706. 10.3390/ijms23052706. PMC8911215 35269848

[83] Zmuidinaite R, Sharara FI, Iles RK. Current advancements in noninvasive profiling of the embryo culture media secretome. Int J Mol Sci 2021; 22:2513. 10.3390/ijms22052513. PMC7959312 33802374

[84] Stigliani S, Anserini P, Venturini PL, Scaruffi P. Mitochondrial DNA content in embryo culture medium is significantly associated with human embryo fragmentation. Hum Reprod 2013; 28:2652–2660. 10.1093/humrep/det314. 23887072

[85] Hammond ER, Shelling AN, Cree LM. Nuclear and mitochondrial DNA in blastocoele fluid and embryo culture medium: evidence and potential clinical use. Hum Reprod 2016; 31:1653–1661. 10.1093/humrep/dew132. 27270971

[86] Palini S, Galluzzi L, de Stefani S, Bianchi M, Wells D, Magnani M, Bulletti C. Genomic DNA in human blastocoele fluid. Reprod Biomed Online 2013; 26:603–610. 10.1016/j.rbmo.2013.02.012. 23557766

[87] Tobler KJ, Zhao Y, Ross R, Benner AT, Xu X, du L, Broman K, Thrift K, Brezina PR, Kearns WG. Blastocoel fluid from differentiated blastocysts harbors embryonic genomic material capable of a whole-genome deoxyribonucleic acid amplification and comprehensive chromosome microarray analysis. Fertil Steril 2015; 104:418–425. 10.1016/j.fertnstert.2015.04.028. 26006737

[88] Vera-Rodriguez M, Diez-Juan A, Jimenez-Almazan J, Martinez S, Navarro R, Peinado V, Mercader A, Meseguer M, Blesa D, Moreno I, Valbuena D, Rubio C, et al. Origin and composition of cell-free DNA in spent medium from human embryo culture during preimplantation development. Hum Reprod Oxf Engl 2018; 33:745–756. 10.1093/humrep/dey028. 29471395

[89] Xu J, Fang R, Chen L, Chen D, Xiao JP, Yang W, Wang H, Song X, Ma T, Bo S, Shi C, Ren J, et al. Noninvasive chromosome screening of human embryos by genome sequencing of embryo culture medium for in vitro fertilization. Proc Natl Acad Sci U S A 2016; 113:11907–11912. 10.1073/pnas.1613294113. PMC5081593 27688762

[90] Handayani N, Aubry D, Boediono A, Bowolaksono A, Sini I, Haq NMD, Sirait B, Periastiningrum G, Mutia K, Wiweko B. Non-invasive pre-implantation genetic testing’s reliability for aneuploidy using cell-free DNA in embryo culture media. J Gynecol Obstet Hum Reprod 2024; 53:102808. 10.1016/j.jogoh.2024.102808. 38825167

[91] Huang L, Bogale B, Tang Y, Lu S, Xie XS, Racowsky C. Noninvasive preimplantation genetic testing for aneuploidy in spent medium may be more reliable than trophectoderm biopsy. Proc Natl Acad Sci U S A 2019; 116:14105–14112. 10.1073/pnas.1907472116. PMC6628824 31235575

[92] Abdellatif SFA, Hussein AAA, Youssef AA. Mitochondrial DNA in day 3 culture medium as a molecular biomarker of human embryo fragmentation, aneuploidy, and implantation competency. Middle East Fertil Soc J 2025; 30:17. 10.1186/s43043-025-00230-z.

[93] Stigliani S, Persico L, Lagazio C, Anserini P, Venturini PL, Scaruffi P. Mitochondrial DNA in day 3 embryo culture medium is a novel, non-invasive biomarker of blastocyst potential and implantation outcome. MHR Basic Sci Reprod Med 2014; 20:1238–1246. 10.1093/molehr/gau086. 25232043

[94] Wu H, Ding C, Shen X, Wang J, Li R, Cai B, Xu Y, Zhong Y, Zhou C. Medium-based noninvasive preimplantation genetic diagnosis for human α-thalassemias-SEA. Medicine (Baltimore) 2015; 94:e669. 10.1097/MD.0000000000000669. PMC4554004 25816038

[95] Hammond ER, McGillivray BC, Wicker SM, Peek JC, Shelling AN, Stone P, Chamley LW, Cree LM. Characterizing nuclear and mitochondrial DNA in spent embryo culture media: genetic contamination identified. Fertil Steril 2017; 107:220–228.e5. 10.1016/j.fertnstert.2016.10.015. 27865449

[96] Bakalova DN, Navarro-Sánchez L, Rubio C. Non-invasive preimplantation genetic testing. Genes 2025; 16:552. 10.3390/genes16050552. PMC12111310 40428374

[97] Yin B, Zhang H, Xie J, Wei Y, Zhang C, Meng L. Validation of preimplantation genetic tests for aneuploidy (PGT-A) with DNA from spent culture media (SCM): concordance assessment and implication. Reprod Biol Endocrinol 2021; 19:41. 10.1186/s12958-021-00714-3. PMC7936457 33673853

[98] Rubio C, Navarro-Sánchez L, García-Pascual CM, Ocali O, Cimadomo D, Venier W, Barroso G, Kopcow L, Bahçeci M, Kulmann MIR, López L, de la Fuente E, et al. Multicenter prospective study of concordance between embryonic cell-free DNA and trophectoderm biopsies from 1301 human blastocysts. Am J Obstet Gynecol 2020; 223:751.e1–751.e13. 10.1016/j.ajog.2020.04.035. 32470458

[99] May-Panloup P, Boguenet M, El Hachem H, Bouet P-E, Reynier P. Embryo and its mitochondria. Antioxidants 2021; 10:139. 10.3390/antiox10020139. PMC7908991 33498182

[100] Podolak A, Woclawek-Potocka I, Lukaszuk K. The role of mitochondria in human fertility and early embryo development: what can we learn for clinical application of assessing and improving mitochondrial DNA? Cells 2022; 11:797. 10.3390/cells11050797. PMC8909547 35269419

[101] Cecchino GN, Seli E, Alves Da Motta EL, García-Velasco JA. The role of mitochondrial activity in female fertility and assisted reproductive technologies: overview and current insights. Reprod Biomed Online 2018; 36:686–697. 10.1016/j.rbmo.2018.02.007. 29598846

[102] Reynier P, May-Panloup P, Chretien MF, Morgan CJ, Jean M, Savagner F, Barriere P, Malthiery Y. Mitochondrial DNA content affects the fertilizability of human oocytes. Mol Hum Reprod 2001; 7:425–429. 10.1093/molehr/7.5.425. 11331664

[103] Diez-Juan A, Rubio C, Marin C, Martinez S, al-Asmar N, Riboldi M, Díaz-Gimeno P, Valbuena D, Simón C. Mitochondrial DNA content as a viability score in human euploid embryos: less is better. Fertil Steril 2015; 104:534–541.e1. 10.1016/j.fertnstert.2015.05.022. 26051102

[104] Teerawongsuwan S, Wiangwised K, Ngampiyakul N, Wanikorn N, Boonnithipaisit P, Khiuhok P, Aekudompong P, Narkwichean A, Pongmayteegul S, Rungsiwiwut R. Relative mtDNA copy number in embryo spent culture medium is not a reliable biomarker of human embryo aneuploidy. Reprod Fertil 2025; 6:e250001. 10.1530/RAF-25-0001. PMC12060771 40243775

[105] Zhang X, Sun Y, Dong X, Zhou J, Sun F, Han T, Lei P, Mao R, Guo X, Wang Q, Li P, Qu T, et al. Mitochondrial DNA and genomic DNA ratio in embryo culture medium is not a reliable predictor for in vitro fertilization outcome. Sci Rep 2019; 9:5378. 10.1038/s41598-019-41801-1. PMC6441050 30926852

[106] Orvieto R, Shimon C, Rienstein S, Jonish-Grossman A, Shani H, Aizer A. Do human embryos have the ability of self-correction? Reprod Biol Endocrinol 2020; 18:98. 10.1186/s12958-020-00650-8. PMC7539487 33023576

[107] Gross N, Kropp J, Khatib H. MicroRNA signaling in embryo development. Biology 2017; 6:34. 10.3390/biology6030034. PMC5617922 28906477

[108] Siristatidis C, Vogiatzi P, Brachnis N, Liassidou A, Iliodromiti Z, Bettocchi S, Chrelias C. MicroRNAs in assisted reproduction and their potential role in IVF failure. In Vivo 2015; 29:169–175. 25792643

[109] Abu-Halima M, Khaizaran ZA, Ayesh BM, Fischer U, Khaizaran SA, al-Battah F, Hammadeh M, Keller A, Meese E. MicroRNAs in combined spent culture media and sperm are associated with embryo quality and pregnancy outcome. Fertil Steril 2020; 113:970–980.e2. 10.1016/j.fertnstert.2019.12.028. 32222254

[110] Wang S, Chen L, Zhu Y, Jiang W. Characterization of microRNAs in spent culture medium associated with human embryo quality and development. Ann Transl Med 2021; 9:1648. 10.21037/atm-21-5029. PMC8667117 34988157

[111] Capalbo A, Ubaldi FM, Cimadomo D, Noli L, Khalaf Y, Farcomeni A, Ilic D, Rienzi L. MicroRNAs in spent blastocyst culture medium are derived from trophectoderm cells and can be explored for human embryo reproductive competence assessment. Fertil Steril 2016; 105:225–235.e3. 10.1016/j.fertnstert.2015.09.014. 26453979

[112] Almutlaq A, Viñals Gonzalez X, SenGupta S. Differentially expressed microRNAs in aneuploid preimplantation blastocysts: a systematic review. Front Reprod Health 2024; 6:1370341. 10.3389/frph.2024.1370341. PMC10973143 38550247

[113] Esmaeilivand M, Ghasemzadeh A, Niknafs B, Fattahi A. Association of trophectoderm mRNAs and microRNAs with chromosomal aneuploidy of embryo. Reprod Sci Thousand Oaks Calif 2024; 31:1028–1033. 10.1007/s43032-023-01381-y. 37964074

[114] Rosenbluth EM, Shelton DN, Wells LM, Sparks AET, Van Voorhis BJ. Human embryos secrete microRNAs into culture media—a potential biomarker for implantation. Fertil Steril 2014; 101:1493–1500. 10.1016/j.fertnstert.2014.01.058. 24786747

[115] Acuña-González RJ, Morales-Hernández FV, López-Canales JS, Lozano-Cuenca J, Osorio-Caballero M, Flores-Herrera H. MiR-191-5p is upregulated in culture media of implanted human embryo on day three of development. Research Square 2020; 22:2513. 10.21203/rs.3.rs-122465/v1. PMC8278618 34256783

[116] Acuña-González RJ, Olvera-Valencia M, López-Canales JS, Lozano-Cuenca J, Osorio-Caballero M, Flores-Herrera H. MiR-191-5p is upregulated in culture media of implanted human embryo on day fifth of development. Reprod Biol Endocrinol 2021; 19:109. 10.1186/s12958-021-00786-1. PMC8278618 34256783

[117] Sánchez-Ribas I, Diaz-Gimeno P, Quiñonero A, Ojeda M, Larreategui Z, Ballesteros A, Domínguez F. NGS analysis of human embryo culture media reveals miRNAs of extra embryonic origin. Reprod Sci Thousand Oaks Calif 2019; 26:214–222. 10.1177/1933719118766252. 29587610

[118] Kamijo S, Hamatani T, Sasaki H, Suzuki H, Abe A, Inoue O, Iwai M, Ogawa S, Odawara K, Tanaka K, Mikashima M, Suzuki M, et al. MicroRNAs secreted by human preimplantation embryos and IVF outcome. Reprod Biol Endocrinol RBE 2022; 20:130. 10.1186/s12958-022-00989-0. PMC9425991 36042522

[119] Galluzzi L, Palini S, Stefani SD, Andreoni F, Primiterra M, Diotallevi A, Bulletti C, Magnani M. Extracellular embryo genomic DNA and its potential for genotyping applications. Future Sci OA 2015; 1:FSO62. 10.4155/fso.15.62. PMC5137924 28031914

[120] Zagers MS, Laverde M, Goddijn M, de Groot JJ, Schrauwen FAP, Vaz FM, Mastenbroek S. The composition of commercially available human embryo culture media. Hum Reprod 2024; 40:30–40. 10.1093/humrep/deae248. PMC11700899 39585967

[121] Mantikou E, Youssef MAFM, van Wely M, van der Veen F, al-Inany HG, Repping S, Mastenbroek S. Embryo culture media and IVF/ICSI success rates: a systematic review. Hum Reprod Update 2013; 19:210–220. 10.1093/humupd/dms061. 23385469

[122] Montag M, Liebenthron J, Köster M. Which morphological scoring system is relevant in human embryo development? Placenta 2011; 32:S252–S256. 10.1016/j.placenta.2011.07.009. 21782239

[123] Bhide P, Chan DYL, Lanz D, Alqawasmeh O, Barry E, Baxter D, Gonzalez Carreras F, Choudhury Y, Cheong Y, Chung JPW, Collins B, Cong L, et al. Clinical effectiveness and safety of time-lapse imaging systems for embryo incubation and selection in in-vitro fertilisation treatment (TILT): a multicentre, three-parallel-group, double-blind, randomised controlled trial. Lancet 2024; 404:256–265. 10.1016/S0140-6736(24)00816-X. 39033010

[124] AlSaad R, Abusarhan L, Odeh N, Abd-alrazaq A, Choucair F, Zegour R, Ahmed A, Aziz S, Sheikh J. Deep learning applications for human embryo assessment using time-lapse imaging: scoping review. Front Reprod Health 2025; 7:1549642. 10.3389/frph.2025.1549642. PMC12011738 40264925

[125] Armstrong S, Bhide P, Jordan V, Pacey A, Farquhar C. Time-lapse systems for embryo incubation and assessment in assisted reproduction. Cochrane Database Syst Rev 2018; 2018:CD011320. 10.1002/14651858.CD011320.pub3. PMC6494546 29800485

[126] Kirkegaard K, Kesmodel US, Hindkjær JJ, Ingerslev HJ. Time-lapse parameters as predictors of blastocyst development and pregnancy outcome in embryos from good prognosis patients: a prospective cohort study. Hum Reprod 2013; 28:2643–2651. 10.1093/humrep/det300. 23900207

[127] Kirkegaard K, Ahlström A, Ingerslev HJ, Hardarson T. Choosing the best embryo by time lapse versus standard morphology. Fertil Steril 2015; 103:323–332. 10.1016/j.fertnstert.2014.11.003. 25527231

[128] Meseguer M, Rubio I, Cruz M, Basile N, Marcos J, Requena A. Embryo incubation and selection in a time-lapse monitoring system improves pregnancy outcome compared with a standard incubator: a retrospective cohort study. Fertil Steril 2012; 98:1481–1489.e10. 10.1016/j.fertnstert.2012.08.016. 22975113

[129] Dal Canto M, Coticchio G, Mignini Renzini M, de Ponti E, Novara PV, Brambillasca F, Comi R, Fadini R. Cleavage kinetics analysis of human embryos predicts development to blastocyst and implantation. Reprod Biomed Online 2012; 25:474–480. 10.1016/j.rbmo.2012.07.016. 22995750

[130] Adamson GD, Abusief ME, Palao L, Witmer J, Palao LM, Gvakharia M. Improved implantation rates of day 3 embryo transfers with the use of an automated time-lapse–enabled test to aid in embryo selection. Fertil Steril 2016; 105:369–375.e6. 10.1016/j.fertnstert.2015.10.030. 26604069

[131] Meng Q, Xu Y, Zheng A, Li H, Ding J, Xu Y, Pu Y, Wang W, Wu H. Noninvasive embryo evaluation and selection by time-lapse monitoring vs. conventional morphologic assessment in women undergoing in vitro fertilization/intracytoplasmic sperm injection: a single-center randomized controlled study. Fertil Steril 2022; 117:1203–1212. 10.1016/j.fertnstert.2022.02.015. 35367059

[132] Kieslinger DC, Vergouw CG, Ramos L, Arends B, Curfs MHJM, Slappendel E, Kostelijk EH, Pieters MHEC, Consten D, Verhoeven MO, Besselink DE, Broekmans F, et al. Clinical outcomes of uninterrupted embryo culture with or without time-lapse-based embryo selection versus interrupted standard culture (SelecTIMO): a three-armed, multicentre, double-blind, randomised controlled trial. Lancet 2023; 401:1438–1446. 10.1016/S0140-6736(23)00168-X. 37004670

[133] Kirkegaard K, Hindkjaer JJ, Grøndahl ML, Kesmodel US, Ingerslev HJ. A randomized clinical trial comparing embryo culture in a conventional incubator with a time-lapse incubator. J Assist Reprod Genet 2012; 29:565–572. 10.1007/s10815-012-9750-x. PMC3370049 22460082

[134] Nakahara T, Iwase A, Goto M, Harata T, Suzuki M, Ienaga M, Kobayashi H, Takikawa S, Manabe S, Kikkawa F, Ando H. Evaluation of the safety of time-lapse observations for human embryos. J Assist Reprod Genet 2010; 27:93–96. 10.1007/s10815-010-9385-8. PMC2842873 20127164

[135] Park H, Bergh C, Selleskog U, Thurin-Kjellberg A, Lundin K. No benefit of culturing embryos in a closed system compared with a conventional incubator in terms of number of good quality embryos: results from an RCT. Hum Reprod Oxf Engl 2015; 30:268–275. 10.1093/humrep/deu316. 25432920

[136] Dimitriadis I, Zaninovic N, Badiola AC, Bormann CL. Artificial intelligence in the embryology laboratory: a review. Reprod Biomed Online 2022; 44:435–448. 10.1016/j.rbmo.2021.11.003. 35027326

[137] Danardono GB, Handayani N, Louis CM, Polim AA, Sirait B, Periastiningrum G, Afadlal S, Boediono A, Sini I. Embryo ploidy status classification through computer-assisted morphology assessment. AJOG Glob Rep 2023; 3:100209. 10.1016/j.xagr.2023.100209. PMC10461251 37645653

[138] Sarker IH . Deep learning: a comprehensive overview on techniques, taxonomy, applications and research directions. SN Comput Sci 2021; 2:420. 10.1007/s42979-021-00815-1. PMC8372231 34426802

[139] Kelleher JD . Deep learning. In: The MIT Press Essential Knowledge Series. Cambridge: The MIT Press; 2019.

[140] VerMilyea M, Hall JMM, Diakiw SM, Johnston A, Nguyen T, Perugini D, Miller A, Picou A, Murphy AP, Perugini M. Development of an artificial intelligence-based assessment model for prediction of embryo viability using static images captured by optical light microscopy during IVF. Hum Reprod 2020; 35:770–784. 10.1093/humrep/deaa013. PMC7192535 32240301

[141] Berntsen J, Rimestad J, Lassen JT, Tran D, Kragh MF. Robust and generalizable embryo selection based on artificial intelligence and time-lapse image sequences. PloS One 2022; 17:e0262661. 10.1371/journal.pone.0262661. PMC8809568 35108306

[142] Enatsu N, Miyatsuka I, An LM, Inubushi M, Enatsu K, Otsuki J, Iwasaki T, Kokeguchi S, Shiotani M. A novel system based on artificial intelligence for predicting blastocyst viability and visualizing the explanation. Reprod Med Biol 2022; 21:e12443. 10.1002/rmb2.12443. PMC8967284 35386375

[143] Salih M, Austin C, Warty RR, Tiktin C, Rolnik DL, Momeni M, Rezatofighi H, Reddy S, Smith V, Vollenhoven B, Horta F. Embryo selection through artificial intelligence versus embryologists: a systematic review. Hum Reprod Open 2023; 2023:hoad031. 10.1093/hropen/hoad031. PMC10426717 37588797

[144] Barnes J, Brendel M, Gao VR, Rajendran S, Kim J, Li Q, Malmsten JE, Sierra JT, Zisimopoulos P, Sigaras A, Khosravi P, Meseguer M, et al. A non-invasive artificial intelligence approach for the prediction of human blastocyst ploidy: a retrospective model development and validation study. Lancet Digit Health 2023; 5:e28–e40. 10.1016/S2589-7500(22)00213-8. PMC10193126 36543475

[145] Chavez-Badiola A, Flores-Saiffe-Farías A, Mendizabal-Ruiz G, Drakeley AJ, Cohen J. Embryo Ranking Intelligent Classification Algorithm (ERICA): artificial intelligence clinical assistant predicting embryo ploidy and implantation. Reprod Biomed Online 2020; 41:585–593. 10.1016/j.rbmo.2020.07.003. 32843306

[146] Blais I, Koifman M, Feferkorn I, Dirnfeld M, Lahav-Baratz S. Improving embryo selection by the development of a laboratory-adapted time-lapse model. FS Sci 2021; 2:176–197. 10.1016/j.xfss.2021.02.001. 35559752

[147] Meseguer M, Herrero J, Tejera A, Hilligsoe KM, Ramsing NB, Remohi J. The use of morphokinetics as a predictor of embryo implantation. Hum Reprod 2011; 26:2658–2671. 10.1093/humrep/der256. 21828117

[148] Kragh MF, Rimestad J, Berntsen J, Karstoft H. Automatic grading of human blastocysts from time-lapse imaging. Comput Biol Med 2019; 115:103494. 10.1016/j.compbiomed.2019.103494. 31630027

[149] Ortiz JA, Morales R, Lledó B, Vicente JA, González J, García-Hernández EM, Cascales A, ten J, Bernabeu A, Bernabeu R. Application of machine learning to predict aneuploidy and mosaicism in embryos from in vitro fertilization cycles. AJOG Glob Rep 2022; 2:100103. 10.1016/j.xagr.2022.100103. PMC9574883 36275401

[150] Diakiw SM, Hall JMM, VerMilyea MD, Amin J, Aizpurua J, Giardini L, Briones YG, Lim AYX, Dakka MA, Nguyen TV, Perugini D, Perugini M. Development of an artificial intelligence model for predicting the likelihood of human embryo euploidy based on blastocyst images from multiple imaging systems during IVF. Hum Reprod 2022; 37:1746–1759. 10.1093/humrep/deac131. PMC9340116 35674312

[151] Chen K, Li Z, Huang G, Guo J. Embryo-Net: a blastocyst image segmentation network based on spatial modeling to resolve the challenge of textural similarity between the TE and ICM. Neurocomputing 2025; 638:130153. 10.1016/j.neucom.2025.130153.

[152] Filho ES, Noble JA, Poli M, Griffiths T, Emerson G, Wells D. A method for semi-automatic grading of human blastocyst microscope images. Hum Reprod 2012; 27:2641–2648. 10.1093/humrep/des219. 22736327

[153] Wyroba J, Kuczyńska A, Kasperkowicz K, Kostarczyk K, Kordowitzki P, Kochan J. Beyond spherical: unveiling the significance of oval blastocyst morphology on euploidy and implantation success. Cells 2025; 14:1468. 10.3390/cells14181468. PMC12468772 41002433

[154] De Gheselle S, et al. Machine learning for prediction of euploidy in human embryos: in search of the best-performing model and predictive features. Fertil Steril 2022; 117:738–746. 10.1016/j.fertnstert.2021.11.029. 35058042

[155] Yang Y, Wang H, du K, Wang X, Zhao J, Han D, Yang Y, Zang S. Factors associated with patient privacy concerns in AI-based health monitoring devices among nursing students: a cross-sectional study. BMC Nurs 2025; 24:726. 10.1186/s12912-025-03453-7. PMC12210904 40598528

[156] Shoham G, Alexandroni H, Weissman A, Mizrachi Y. Global trends in the use of artificial intelligence (AI) in reproductive medicine: insights from surveys of international fertility specialists. J IVF-Worldw 2025; 3:33–34. 10.46989/001c.140673.

[157] Kim HM, Kang H, Lee C, Park JH, Chung MK, Kim M, Kim NY, Lee HJ. Evaluation of the clinical efficacy and trust in AI-assisted embryo ranking: survey-based prospective study. J Med Internet Res 2024; 26:e52637. 10.2196/52637. PMC11184268 38830209

[158] Montesinos López OA, Montesinos López A, Crossa J. Overfitting, model tuning, and evaluation of prediction performance. In: Multivariate Statistical Machine Learning Methods for Genomic Prediction. Cham: Springer International Publishing, 2022, pp. 109–139. 10.1007/978-3-030-89010-0_4. 36103587

[159] Mould RR, Mackenzie AM, Kalampouka I, Nunn AVW, Thomas EL, Bell JD, Botchway SW. Ultra weak photon emission—a brief review. Front Physiol 2024; 15:1348915. 10.3389/fphys.2024.1348915. PMC10899412 38420619

[160] Bodis J, et al. Detection of ultraweak photon emissions from mouse embryos with implications for assisted reproduction. SSRN 2023; 9:9041. 10.2139/ssrn.4622460.

[161] Berke J, Gulyás I, Bognár Z, Berke D, Enyedi A, Kozma-Bognár V, Mauchart P, Nagy B, Várnagy Á, Kovács K, Bódis J. Unique algorithm for the evaluation of embryo photon emission and viability. Sci Rep 2024; 14:15066. 10.1038/s41598-024-61100-8. PMC11220017 38956113

[162] Van Wijk R, Van Wijk EPA, Van Wietmarschen HA, Greef JVD. Towards whole-body ultra-weak photon counting and imaging with a focus on human beings: a review. J Photochem Photobiol B 2014; 139:39–46. 10.1016/j.jphotobiol.2013.11.014. 24359911

[163] Ahmadi B, Schwarz T, Bartlewski PM. Validation of new proprietary software (r-Algo) for predicting meat chemical composition from ultrasound images of skeletal muscles in live animals: pectoralis major muscles of broiler chickens. J Anim Feed Sci 2024; 33:368–379. 10.22358/jafs/186021/2024.

[164] Bartlewski J, Oliveira NZ, Fryc K, Murawski M, Ahmadi B, Bartlewski PM. Relationships of phototextural characteristics of ovine presumptive zygotes to their developmental potential in vitro. Biol Reprod 2025; 113:787–793. 10.1093/biolre/ioaf135. PMC12527285 40576338

[165] Farid I, Hamid N, Bartlewski J, Singh S, Mirshahi A, Bartlewski PM, Ahmadi B. Chemical profiling of testicular parenchyma in rams using an exploration of echointensity bands and the novel r-Algo computer algorithm increasing precision and accuracy of ultrasound image analyses. Reprod Biol 2025; 25:101021. 10.1016/j.repbio.2025.101021. 40288041

[166] Abass M, Bartlewski J, Bartlewski PM, Kochan J, Murawski M, Singh S, Wyroba J. Algorithmic image analysis reveals differences between aneuploid and euploid ICSI-derived human blastocysts. Poster presentation at: *The Southern Ontario Reproductive Biology Society* 52nd Annual Meeting. ON, Canada: Hamilton; May 30th, 2025.

[167] Minasi MG, Colasante A, Riccio T, Ruberti A, Casciani V, Scarselli F, Spinella F, Fiorentino F, Varricchio MT, Greco E. Correlation between aneuploidy, standard morphology evaluation and morphokinetic development in 1730 biopsied blastocysts: a consecutive case series study. Hum Reprod 2016; 31:2245–2254. 10.1093/humrep/dew183. 27591227

[168] Khajehoseini F, Noormohammadi Z, Eftekhari-Yazdi P, Gourabi H, Pazhoomand R, Hosseinishenatal S, Bazrgar M. Correlation among blastocoel fluid SNA level, apoptotic genes expressiona dn preimplantation aneuploidy. *Reprod Fertil* 2025; 6:e240097. 10.1530/RAF-24-0097. PMC11825185 39869096

[169] Lee I-W, Tazehkand AP, Sha Z-Y, Adhikari D, Carroll J. An aggregated mitochondrial distribution in preimplantation embryos disrupts nuclear morphology, function, and developmental potential. Proc Natl Acad Sci 2024; 121:e2317316121. 10.1073/pnas.2317316121. PMC11228517 38917013

[170] Wang M, Gao L, Yang Q, Long R, Zhang Y, Jin L, Zhu L. Does smooth endoplasmic reticulum aggregation in oocytes impact the chromosome aneuploidy of the subsequent embryos? A propensity score matching study. J Ovarian Res 2023; 16:59. 10.1186/s13048-023-01135-z. PMC10037775 36959673

[171] Capalbo A, Romanelli V, Patassini C, Poli M, Girardi L, Giancani A, Stoppa M, Cimadomo D, Ubaldi FM, Rienzi L. Diagnostic efficacy of blastocoel fluid and spent media as sources of DNA for preimplantation genetic testing in standard clinical conditions. Fertil Steril 2018; 110:870–879.e5. 10.1016/j.fertnstert.2018.05.031. 30316433

[172] Munné S, Horcajadas JA, Seth-Smith ML, Perugini M, Griffin DK. Non-invasive selection for euploid embryos: prospects and pitfalls of the three most promising approaches. Reprod Biomed Online 2025; 51:105077. 10.1016/j.rbmo.2025.105077. 40934618

