# Supplementary material for: “Old and new” frontiers in embryo viability assessment: current practices and introduction of emerging approaches
Source: Biol Reprod. 2026 Jul 14;115(4):1013–31. doi: 10.1093/biolre/ioag147 (PMC13669403; doi:10.1093/biolre/ioag147)
Supplement: Supplementary file 1 [file supplementary_material_ioag147.zip › supplementary_fig_caption_ioag147.docx]

**Supplemental Fig. 1.** The pronuclear grading scheme developed by Tesarik and Greco [13] classifies and evaluates the morphology of zygotes after fertilization. This scheme categorizes zygotes into six patterns, from 0 to 5, based on the size, location, and polarization of the pronuclei. Pattern 0 is defined as having the same number of small nucleolar precursor bodies (NPBs) distributed in the nucleus or large NPBs with polar distribution between the two pronuclei. Patterns 1-5 are the zygotes with non-symmetrical alignments of NPBs. Pattern 0 is considered normal, while patterns 1-5 represent various irregularities in pronuclear morphology.

**Supplemental Fig. 2.** Zygote scoring (Z-score) method developed by Scott [14] evaluates pronuclei and nucleolar precursor bodies (NPBs). Z1 and Z2 scores classify non-separated and similarly shaped pronuclei, by the alignment and number of NPBs. Z3-1 to Z3-4 classify non-separated and similarly shaped pronuclei, by the differences in size, number, and alignment of NPBs. Z4-1 to Z4-3 classify pronuclei based on separation between pronuclei and their size.

**Supplemental Fig. 3.** Pronuclear morphology grading scheme from the ESHRE [16] and Scott [14], which classifies embryos as symmetrical, non-symmetrical, or abnormal. The symmetrical zygotes (grade 1) is described as score Z1 and Z2 [14] which have 2 centrally located pronuclei of similar size, with an equivalent number and similar size of NPBs. The unsymmetrical zygotes (grade B) are zygotes with differing arrangements to that of grade A. The abnormal group (grade C) classifies zygotes with either one or no NPBs.

**Supplemental Fig. 4.** Summary of a scoring system for blastocysts proposed by Gardner and Schoolcraft [33] which assesses the blastocysts’ cavity, inner cell mass (ICM), and trophectoderm (TE). Grades 3BB and above (3BB, 3AB, 3BA, 3AA, 4AA, 4AB, 4BA, 4BB, 5AA, 5AB, 5BA, 5BB, 6AA, 6AB, 6BA, and 6BB; highlighted in grey) are considered good to excellent quality blastocysts, whereas blastocysts with ICM or TE=C (e.g., 3AC, 3BC, 4CB) or those that fail to reach the blastocyst stage (≤2) are non-transferable (poor-quality) embryos.

**Supplemental Fig. 1**

**Supplemental Fig. 2**

**Supplemental Fig. 3**

**Supplemental Fig. 4**

| **Blastocoel expansion and hatching** | | **Inner cell mass** | | **Trophectoderm** | |
| --- | --- | --- | --- | --- | --- |
| **Grade/description** | **Characteristic** | **Grade** | **Characteristic** | **Grade** | **Characteristic** |
| **1/Early blastocyst** | Blastocoel <1/2 embryo | **A** | Many tightly packed cells (excellent) | **A** | Many cohesive epithelial cells (excellent) |
| **2/Blastocyst** | Blastocoel >1/2 embryo | **B** | Several loosely grouped cells (good) | **B** | Few, loosely cohesive cells (good) |
| **3/Full blastocyst** | Blastocoel fills entire embryo | **C** | Few or disorganized cells (poor) | **C** | Very few, large, or unevenly distributed cells (poor) |
| **4/Expanded blastocyst** | Blastocoel > early embryo volume, zona pellucida thinning |  |  |  |  |
| **5/Hatching blastocyst** | Trophectoderm starting to break through zona pellucida |  |  |  |  |
| **6/Hatched blastocyst** | Blastomeres fully escaped from zona pellucida |  |  |  |  |
