# Supplementary material for: “Old and new” frontiers in embryo viability assessment: current practices and introduction of emerging approaches
Source: Biol Reprod. 2026 Jul 14;115(4):1013–31. doi: 10.1093/biolre/ioag147 (PMC13669403; doi:10.1093/biolre/ioag147)
Supplement: Supplementary file 1 [file supplementary_material_ioag147.zip › S_S_sup_figure_4_ioag147.pdf]

Blastocoel expansion and hatching		Inner cell mass		Trophectoderm	
**Grade/description**	**Characteristic**	**Grade**	**Characteristic**	**Grade**	**Characteristic**
**1/Early blastocyst**	*Blastocoel <1/2 embryo*	**A**	*Many tightly packed cells (excellent)*	**A**	*Many cohesive epithelial cells (excellent)*
**2/Blastocyst**	*Blastocoel >1/2 embryo*	**B**	*Several loosely grouped cells (good)*	**B**	*Few, loosely cohesive cells (good)*
**3/Full blastocyst**	*Blastocoel fills entire embryo*	**C**	*Few or disorganized cells (poor)*	**C**	*Very few, large, or unevenly distributed cells (poor)*
**4/Expanded blastocyst**	*Blastocoel > early embryo volume, zona pellucida thinning*				
**5/Hatching blastocyst**	*Trophectoderm starting to break through zona pellucida*				
**6/Hatched blastocyst**	*Blastomeres fully escaped from zona pellucida*				
