# Supplementary material for: “Old and new” frontiers in embryo viability assessment: current practices and introduction of emerging approaches
Source: Biol Reprod. 2026 Jul 14;115(4):1013–31. doi: 10.1093/biolre/ioag147 (PMC13669403; doi:10.1093/biolre/ioag147)
Supplement: Supplementary file 1 [file supplementary_material_ioag147.zip › S_S_sup_fig_1_ioag147.pdf]

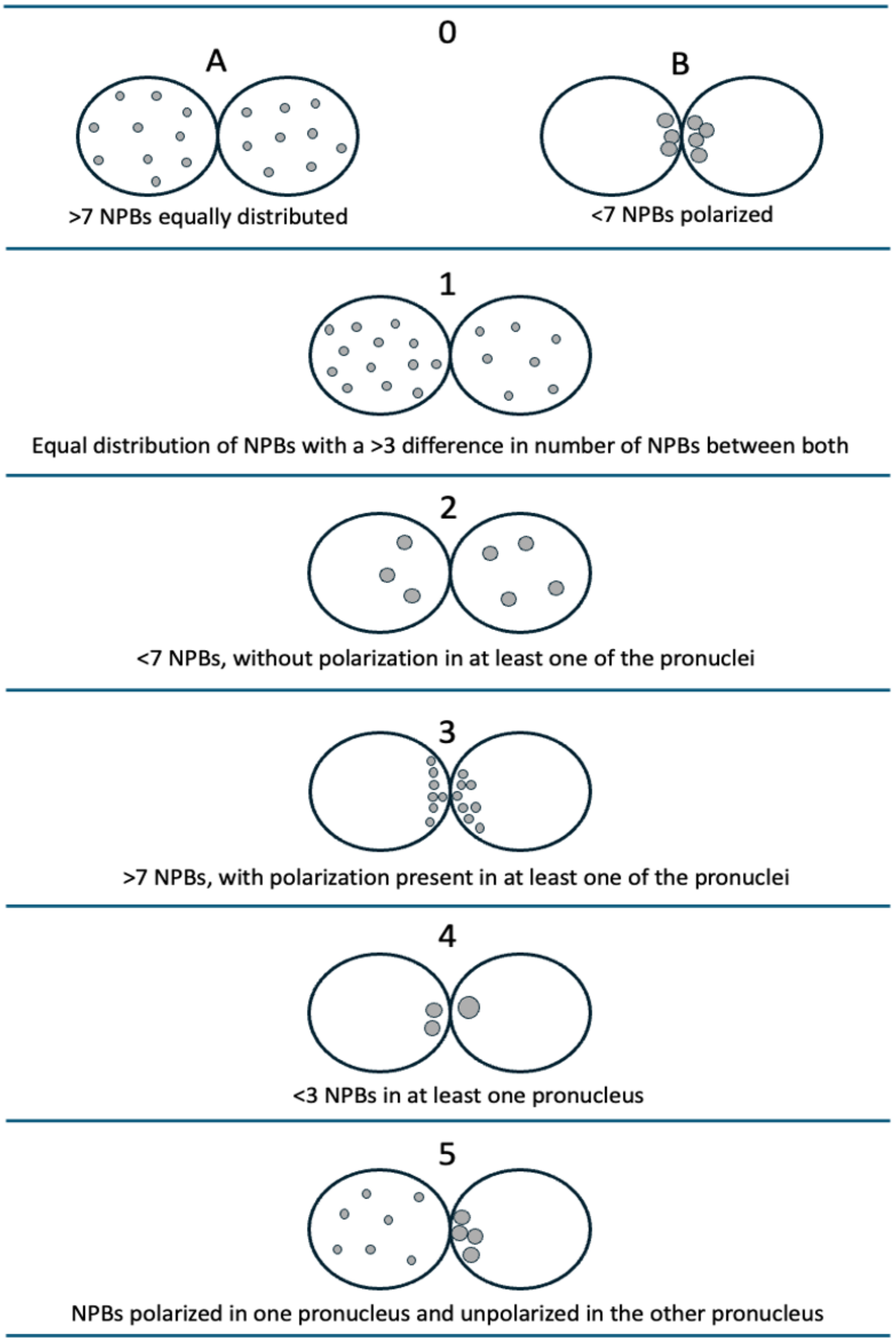
0
A
B
>7 NPBs equally distributed
<7 NPBs polarized
1
Equal distribution of NPBs with a >3 difference in number of NPBs between both
2
<7 NPBs, without polarization in at least one of the pronuclei
3
>7 NPBs, with polarization present in at least one of the pronuclei
4
<3 NPBs in at least one pronucleus
5
NPBs polarized in one pronucleus and unpolarized in the other pronucleus
