# Supplementary material for: “Old and new” frontiers in embryo viability assessment: current practices and introduction of emerging approaches
Source: Biol Reprod. 2026 Jul 14;115(4):1013–31. doi: 10.1093/biolre/ioag147 (PMC13669403; doi:10.1093/biolre/ioag147)
Supplement: Supplementary file 1 [file supplementary_material_ioag147.zip › S_S_sup_fig_2_ioag147.pdf]

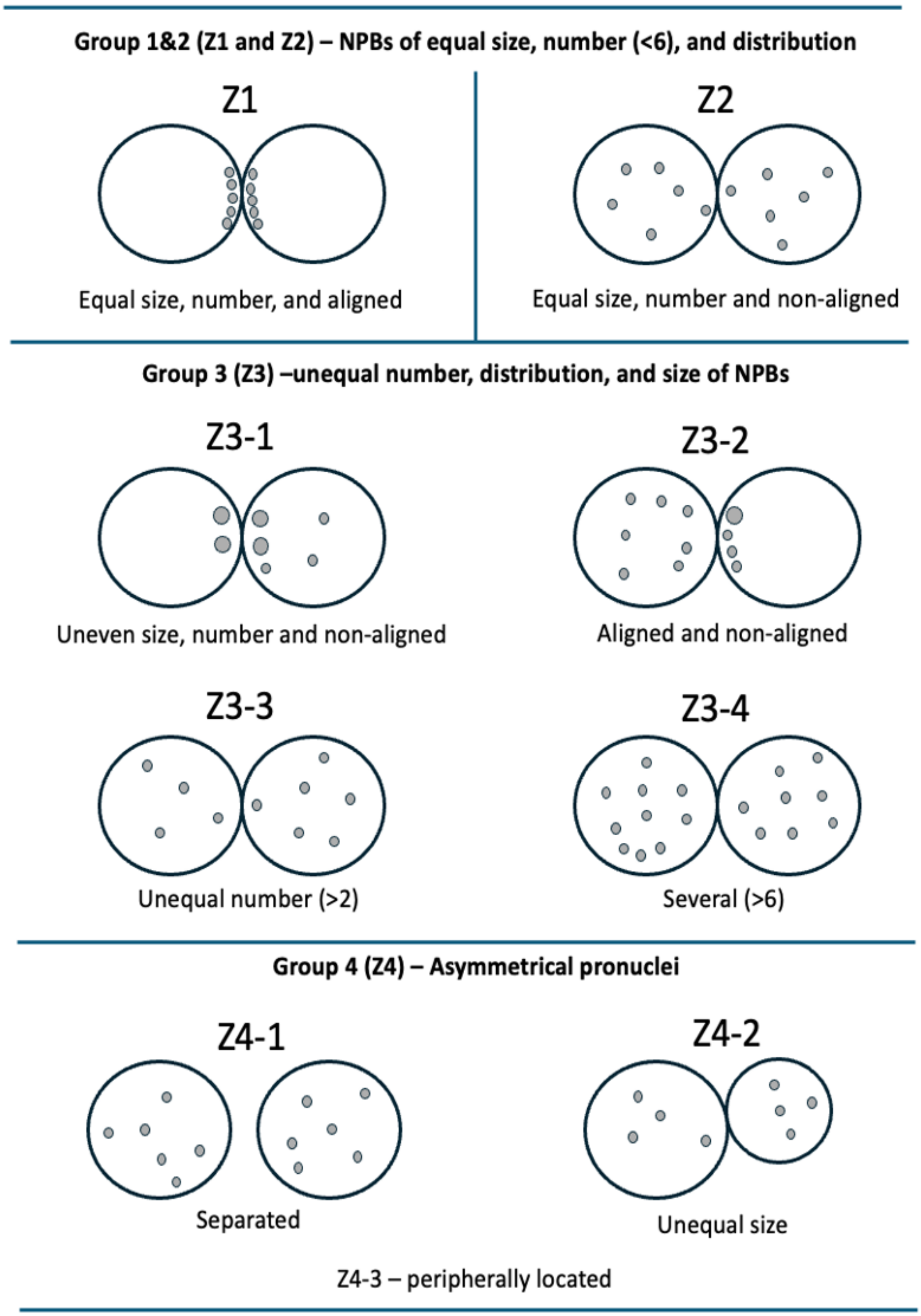
Group 1&2 (Z1 and Z2) – NPBs of equal size, number (<6), and distribution
Z1
Equal size, number, and aligned
Z2
Equal size, number and non-aligned
Group 3 (Z3) –unequal number, distribution, and size of NPBs
Z3-1
Uneven size, number and non-aligned
Z3-2
Aligned and non-aligned
Z3-3
Unequal number (>2)
Z3-4
Several (>6)
Group 4 (Z4) – Asymmetrical pronuclei
Z4-1
Separated
Z4-2
Unequal size
Z4-3 – peripherally located
