# Supplementary material for: “Old and new” frontiers in embryo viability assessment: current practices and introduction of emerging approaches
Source: Biol Reprod. 2026 Jul 14;115(4):1013–31. doi: 10.1093/biolre/ioag147 (PMC13669403; doi:10.1093/biolre/ioag147)
Supplement: Supplementary file 1 [file supplementary_material_ioag147.zip › S_S_sup_fig_3_ioag147.pdf]

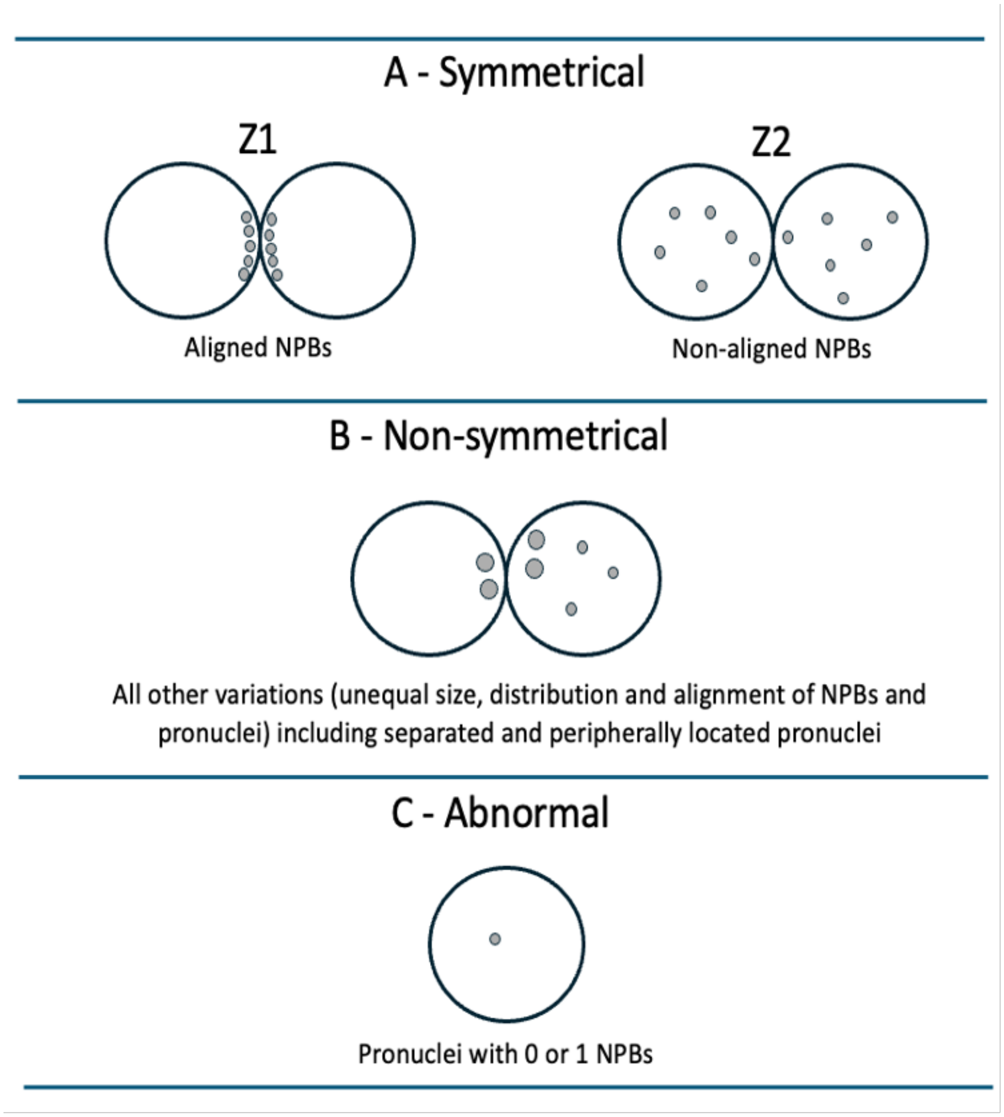
A - Symmetrical
Z1
Aligned NPBs
Z2
Non-aligned NPBs
B - Non-symmetrical
All other variations (unequal size, distribution and alignment of NPBs and pronuclei) including separated and peripherally located pronuclei
C - Abnormal
Pronuclei with 0 or 1 NPBs
